# Identification of potential novel biomarkers to differentiate malignant thyroid nodules with cytological indeterminate

**DOI:** 10.1186/s12885-020-6676-z

**Published:** 2020-03-12

**Authors:** Dandan Wu, Shudong Hu, Yongzhong Hou, Yingying He, Shubai Liu

**Affiliations:** 1 Institute of Life Sciences, Jiangsu University, 301 Xuefu Road, JinKou District, Zhenjiang, 212013 PR China; 2 Department of Radiology, Affiliated Renmin Hospital of Jiangsu University, Zhenjiang, Jiangsu PR China

**Keywords:** Papillary thyroid carcinoma, Biomarker, Thyroid nodules, Biomarker, Fine-needle aspiration biopsy, WGCNA

## Abstract

**Background:**

The fine-needle aspiration (FNA) biopsy was broadly applied to clinical diagnostics evaluation for thyroid carcinomas nodule, while companioning with higher uncertainty rate (15~30%) to identify malignancy for cytological indeterminate cases. It is requirement to discover novel molecular biomarkers to differentiate malignant thyroid nodule more precise.

**Methods:**

We employed weighted gene co-expression network analysis (WGCNA) to discover genes significantly associated with malignant histopathology for cytological indeterminate nodules. In addition, identified significantly genes were validated through another independently investigations of thyroid carcinomas patient’s samples via cBioportal and Geipa. The key function pathways of significant genes involving were blast through GenClip.

**Results:**

Twenty-four signature genes were identified significantly related to thyroid nodules malignancy. Furthermore, five novel genes with missense mutation, *FN1* (R534P), *PROS1*((K200I), (Q571K)), *SCEL* (T320S), *SLC34A2*(T688M) and *TENM1* (S1131F), were highlighted as potential biomarkers to rule out nodules malignancy. It was identified that the key functional pathways involving in thyroid carcinomas.

**Conclusion:**

These results will be helpful to better understand the mechanism of thyroid nodules malignant transformation and characterize the potentially biomarkers for thyroid carcinomas early diagnostics.

## Background

Thyroid cancer is a common malignant neoplasm in worldwide. Recently, the incidence rate of thyroid cancer is rapid raising in the world and becoming the potential threat for public health [1, 2]. It is important to develop early precise diagnostics method and interfere the thyroid neoplasm progress into malignant carcinoma. Up to now, the Fine-needle aspiration (FNA) biopsy is the most accurate and cost-effective tool for thyroid nodules clinical evaluating. It has been strongly recommended by the American Thyroid Association as standardized clinical operation [3–5]. However, about 10~30% cases’ cytological results are indeterminate, and being labelled as indeterminate or suspicious for malignancy. Among these cytological indeterminate cases, majority of patients underwent partial or complete thyroidectomy and checked by histological evaluation. Although the subsequent postsurgical evaluation results reveal only 6~30% cytological indeterminate cases identified as malignant, it made this clinical operation extremely low efficiency and non-specificity while with higher costs [6, 7].

Molecular biomarkers analysis is a powerful adjunct approach to traditional carcinomas pathological evaluation. Multiple molecular markers have been discovered and employed in developing precise diagnostics methods and novel strategies to properly treatment. These biomarkers are generated from gene sequence for gene mutations, gene rearrangements, RNA-based assays, gene expression profiling and immune-histochemistry [8, 9]. As endocrine neoplasm deriving from follicular or para-follicular thyroid cells, thyroid cancer has been reported associated with higher frequency (about 70%) somatic alternation or deletion of genes involving key signaling pathways, such as the mutation of *BRAF* and *RAS* [6, 10], *NTRK1* tyrosine kinases and key effectors mitogen-activated protein kinase (MAPK) signaling pathway [11]. With advanced understanding of thyroid tumor formation, the researches of generic mutation-based biomarkers discovery shifted from single mutation to molecular signatures genes or panels of multiple mutations [12]. According to the previously American Thyroid Association Management Guidelines, a 7-gene molecular biomarkers panel of genetic mutation and rearrangement (7-gene MT), including BRAFV600E, three isoforms of RAS point mutations and translocations of *PAX8/PPARc* and *RET/PTC* genes [6, 13], was recommended to evaluate the residual FNA sample with cytological indeterminate and estimate with high specificity (~ about 90%) [14–16]. Especially, the mutational testing of biomarker genes has been proposed to be a rule-in test with reported higher specificity in clinical practice. Recently, it was reported that the sensitivity of seven-genes mutational panel testing showed huge variation, from 44 to 100% [6, 17]. It is strong suggested that traditional gene mutation panels analysis may not reliably rule out nodules malignancy in some case population. In current, there is no definitively single optimal molecular test that 100% promised to rule-in or rule–out the malignancy in cytology-indeterminate cases [18]. It is necessary to discover novel potential molecular biomarkers to enhance sensitivity and specificity of mutational analysis and precise to rule-in the malignance for cytology indeterminate nodules.

Recently, lacking of long term clinical outcome tracking recording of using molecular markers, there are some controversies over the benefit and limitation of existing molecular markers testing [18]. To enhance the efficiency of thyroid carcinomas patient’s diagnostic, treatment and health management, it is the trend to develop systemic diagnostic strategy and discover novel applicable and specific molecular biomarkers for early diagnostics through analyzing the genetic and expression profiling of thyroid nodules from FNA biopsy [19]. Among multiple computerization methodologies, the Weighted Gene Co-Expression Network Analysis (WGCNA) is considered as one of the most useful approaches to discover gene co-expression network based functional feature through gene expression profiling analysis [20]. Recently, WGCNA is be widely applied to screening the signature genes significantly associated with clinical feature. It is powerful to discover candidate biomarkers for cancer early diagnostics, cancer-associated pathways or therapeutic targets for precise treatment in hepatocellular carcinoma [21], lung cancer [22], endometrial cancer [23] and melanoma [24].

In this study, we employed the WGCNA to analyze gene expression profile of thyroid nodules with cytological indeterminate and aimed to identify the highly connected hub and modules that genes significantly associated with histological malignant thyroid nodule. In addition, we will explore other independently clinical cases through the genetic database to verify the significantly signature genes with genetic changes and discover the key biological pathways significantly associated with malignant thyroid cancer by Genclip pathfinder.

## Methods

### Gene expression data source

**Fig. 1:**
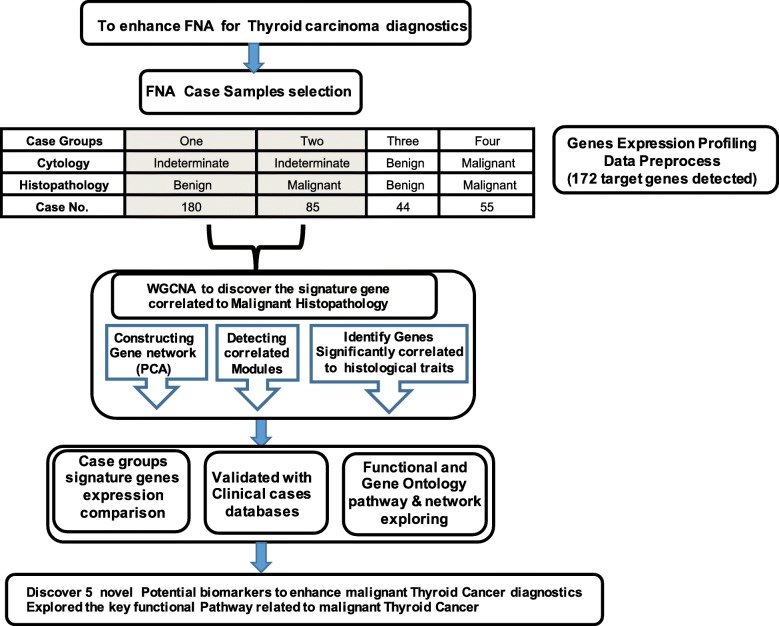
Work flow of the FNA samples with cytology indeterminate for WGCNA

In this study, the dataset applied for data analysis is available in the Gene Expression Omnibus (GEO) repository (https://www.ncbi.nlm.nih.gov/geo/query/acc.cgi?acc=GSE34289) in NCBI, and the platform entry number is GPL14961. This dataset came from the work of Erik et al. (2012) and contained 172 target genes expression data [25]. The samples related information and genes annotated probe-id were transformed into gene symbols and related functional annotations. The related clinical trait annotation was distracted from GSE34289 annotation dataset. Each gene expression value was normalized and performed with log2 transformation. The genes expression profiling of 364 thyroid nodules were split into four groups and 265 samples with cytology indeterminate were selected for further WGCNA analysis as workflow demonstration (Fig. 1).

### Clinical case samples group sorting

According to the clinical cytological / histopathological traits, 364 thyroid nodules samples were split into four groups: Group one (180), cytology-Indeterminate/histopathology-benign; Group two (85), cytology-indeterminate/histopathology-malignant; Group three (44), cytology-benign/histopathology-benign; Group four (55), cytology-malignant /histopathology-benign (Additional file Table S1).

### Construction of weighted gene co-expression network

The WGCNA package of R (version 1.63) was download and setup by following the protocol described previously [26]. The WGCNA package was used to perform various functions in weighted correlation network analysis, including constructing network, detecting module, calculating topological properties, simulating data, visualization, and interfacing with external software [26]. First of all, we have checked data to exclude the sample with excessive missing values and identify outlier microarray samples. After data preprocessing, we applied the principal component analysis (PCA) to double check the data quality. We observed that tumor and normal samples were separated in the PCA plot (Additional file Figure S1), and then we performed hierarchical clustering on the samples to further detect potential outliers. The total 265 samples were used for next step analysis (Fig. 1). We chose the soft threshold β = 7 to construct the co-expression network as the R^2^ reached the peak for the first time when β = 7. The plot of log10(p(k)) versus log10(k) (Additional file Figure S2) indicates that the network is close to a scale-free network by using β = 7, where k is the whole network connectivity and p(k) is the corresponding frequency distribution (Additional file Table S2). When β = 7, the R2 is 0.98, ensuring that the network was close to the scale-free network. After the soft thresholding power β was determined, the Topological Overlap Matrix (TOM) and dissTOM = 1 − TOM were obtained (Additional file Figure S3). After the modules were identified, the T-test was used to calculate the significant *p*-value of candidate genes, and the gene significance (GS) was defined as mediated *p*-value of each gene (GS = lgP). Then, the module significance (MS) were defined as the average GS of all the genes involved in the module. In general, the module with the highest MS among all the selected modules will be considered as the one associated with disease. In addition, we also calculated the relevance between the clinical feature (histopathology) of modules and phenotypes to identify the most relevant module. The hierarchical clustering analysis was used to identify gene modules and color to indicate modules, which is a cluster of densely interconnected genes in terms of co-expression (Additional file Figure S4). For genes that are not assigned to any of the modules, WGCNA places them into a grey module as not co-expressed (Additional file Table S3). The module eigengene (ME) of a module is defined as the first principal component of the module and represents the overall expression level of the module. To identify modules that were significantly associated with the traits of histology, age and gender status, we correlated the MEs (i.e. the first principle component of a module) [27] with clinical traits and searched the most significant associations. A hierarchical clustering of MEs was performed to study the correlations among the modules. we used the linear mixed-effects model (eq. (4)) for testing the association of a module to the histology determinate tumor status [26].

### Exploring the clinical cancer cases databases

Genes significance associated with histology feature of malignant thyroid nodule were blast in the cBioportal Cancer Genomics dataset with independent cases and verified the association of thyroid carcinoma’s patient’s cases to public [28, 29]. In addition, we blast Gene Expression Profiling Interactive Analysis databases (http://gepia.cancer-pku.cn) to validate the expression of these five biomarker candidates.

### Validations of signature genes expression

The validations of significant genes were performed by comparison of expression level among the thyroid nodule case groups and blast in TCGA (The Cancer Genome Atlas) database with independent cases. The case group with cytology indeterminate/histopathology benign was used as the benchmark. The individual gene expression in each group are presented as means ± standard error of the mean (SEM) that represent distribution of group cases. The expression level comparison was used the fold change ratio to quantitatively analyze. The Significance of differences for the values were determined using the student t-test with the Prism software (GraphPad Software, Inc. San Diego, CA). A *P* value < 0.001 was setup as significant difference standard.

### GO and pathway enrichment analysis

We utilized GenCLiP 2.0 tool to collect the correlated Gene Ontology (GO) functional clustering and pathway enrichment analyses for the genes significance in blue module, which is powerful to discovery the abnormal pathway or key components related to certain diseases [30]. The *P* value < 0.05 was setup as the significantly cut-off criterion.

## Results

### Identified gene modules correlation with histological traits

**Fig. 2:**
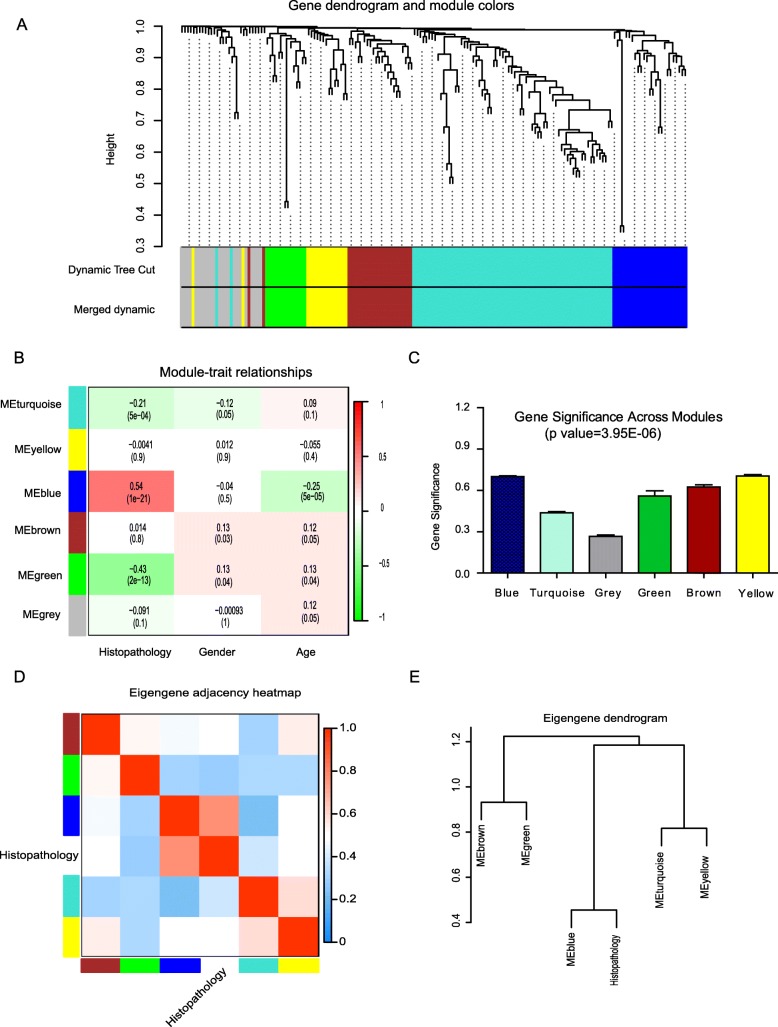
Gene dendrogram and module cluster for Histopathological feature. **a** Clustering dendrogram of genes, with dissimilarity based on topological overlap, is merged with assigned module colors and the original module colors. **b** The correlation of Module-clinical traits. Each row corresponds to a module; each column corresponds to a clinical trait feature. Each cell contains the test statistic value and its corresponding *p* value from the linear mixed-effects model. Network of eigengene represents the relationships among the modules and the histological traits. **c** There are total 6 Module memberships vs. gene significance cluster for histopathology trait. Module membership vs gene significance is correlating to thyroid nodule histopathological status. Panel **d** shows a hierarchical clustering dendrogram of the eigengenes in which the dissimilarity of eigengenes (EI, EJ is given by 1 − cor(EI, EJ). The heatmap in panel (**e**) shows the eigengene adjacency (AI J = (1 + cor (EI, EJ))/2)

In this study, we applied WGCNA to investigate the relationship between gene expression profiling of FNA thyroid nodules with cytology indeterminate (265 cases, group one and group two, Additional file Table S1) and clinical traits-histopathology, age and gender. After using a dynamic tree cutting algorithm, we identified 6 distinct co-expression modules (Fig. 2a), including Blue (24), Turquoise (66), Green (14), Brown (23), Yellow (15) and Grey (29) modules containing with varied different number genes. There are three MEs, Blue, Green and Turquoise, highly significantly correlated to histopathology trait based on the hierarchical clustering analysis (Fig. 2b), and Blue is positive correlated with histopathology trait. Through calculation of the linear mixed-effects model, the turquoise module (t-value = − 0.21, *P* value = 0.004), blue module (t-value = 0.54, *P* value = 1e− 21) and the green module (t-value = − 0.43, *P* value = 2e− 13) are identified significantly associated with malignant thyroid nodule status (Fig. 2c). The blue module, containing 24 genes (Additional file Table S4), is the most significant module (*P* value = 1e− 21) associated with thyroid nodule malignant histopathology feature, while green and turquoise module are negative correlated with malignant feature and do not discuss. The 29 uncorrelated genes were assigned into a grey module, which was ignored in the following analysis (Fig. 2b, and Additional file Table S3).

### Enriched genes significance related to histological feature

Compared the MS among the modules (Fig. 2c), the results showed that the Blue module is the highest relevance and positive correlated to histopathology malignant status (cor = 0.77, *P* value = 6.8e− 06). For each gene contained in a module, we plotted the scatter figure of multiple module memberships (MM) against the GS (Additional file Figure S5A-E). In the WGCNA, the module membership (MM): MM(i) = cor (xi, ME) is defined to measure the importance of the gene within the module. The greater absolute value of MM(i), gene i is more important the in the module. The GS in the blue module is highly correlated with MM, indicating that Gene is significantly associated with malignant histological feature (Fig. 2d, *P* value = 6.8e− 06). The genes significance is also the important element of the Blue module (*P* value = 3.95E-06, Fig. 2e) and listed (Additional file Table S4). The heatmap plot is depicted of topological overlap in the gene network (Additional file Figure S6).

### Validated significant genes through cBioPortal database

**Fig. 3:**
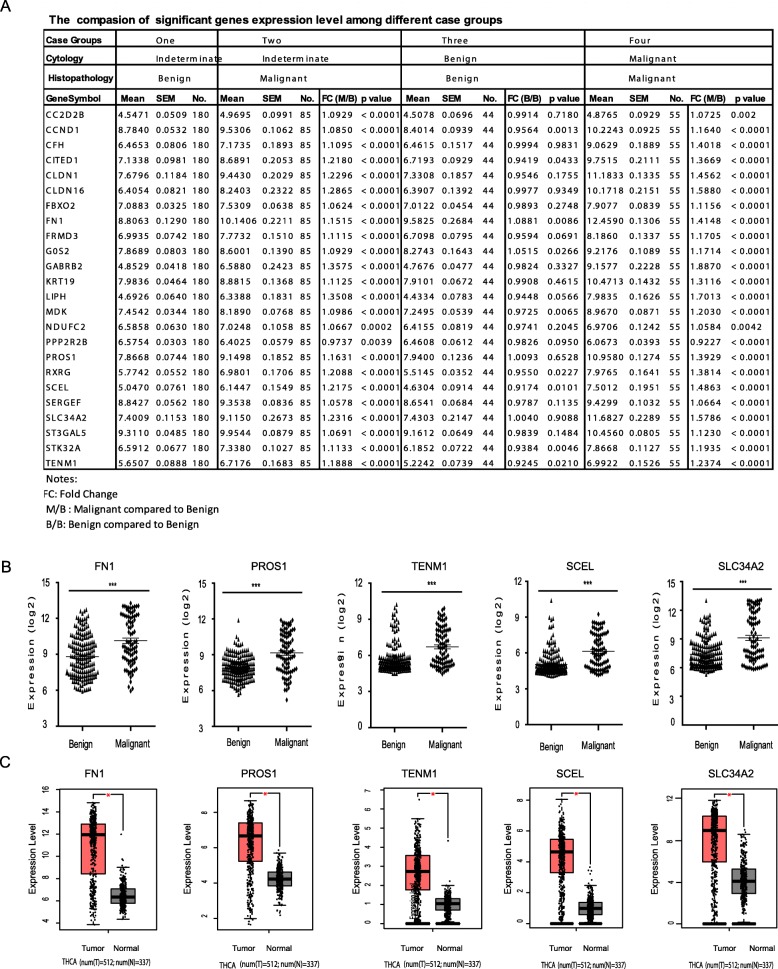
Validation of the gene expression levels of novel biomarkers between histopathological benign and malignant. **a** Expression comparison of 24 signature genes among benign and malignant cases groups; **b** Validation the expression level of potential biomarkers of FN1, TENM1, SCEL, SCL34A2, PROS1. **c** Validation based on TCGA data via GEPIA, including FN1, TENM1, SCEL, SCL34A2, PROS (***, represent *p* value < 0.0001; *, represents *p* value < 0.01)

Compared with cytology-indeterminate/histopathology-benign group as the benchmark, the 23 signature genes showed significant higher expression (Fold change > 1.0, *P* value < 0.001) in the cytology-indeterminate/histopathology-malignant group cases, while only PPP2R2B with lower expression (*P* value = 0.0039, Fig. 3a). In the negative case group, with double cytology/histopathology benign, although 12 genes were lower expression (0.92 < FC < 0.98, *P* value > 0.001), 10 genes were close to equal expression (*CC2D2B, CFH, CLDN16, FBXO2, GABRB2, KRT19, PPP2R2B, ST3GAL5, PROS1, SLC34A2*, 0.98 < FC < 1.01, *P* value > 0.05), and *FN1* (FC = 1.0881, *P* value =0.0086) and *GOS2* (FC = 1.0881, *P* value = 0.0266) indicated higher expression, there are no significantly different expression than control. In the positive case group, with double cytology/histopathology malignant, except *PPP2R2B* with lower (FC = 0.9227, *P* value = 0.0042) and *CC2D2B* with higher expression (FC = 1.0725, *P* value = 0.002), the other 22 genes were significantly higher expression (FC > 1.0, *P* value < 0.0001) than benchmark. Furthermore, the identified 5 potential biomarkers were significantly higher expression (FC > 1.15, *P* value < 0.0001) in both cytology-indeterminate/histopathology-malignant group and double cytology/histopathology malignant group (Fig. 3a&b).

**Fig. 4:**
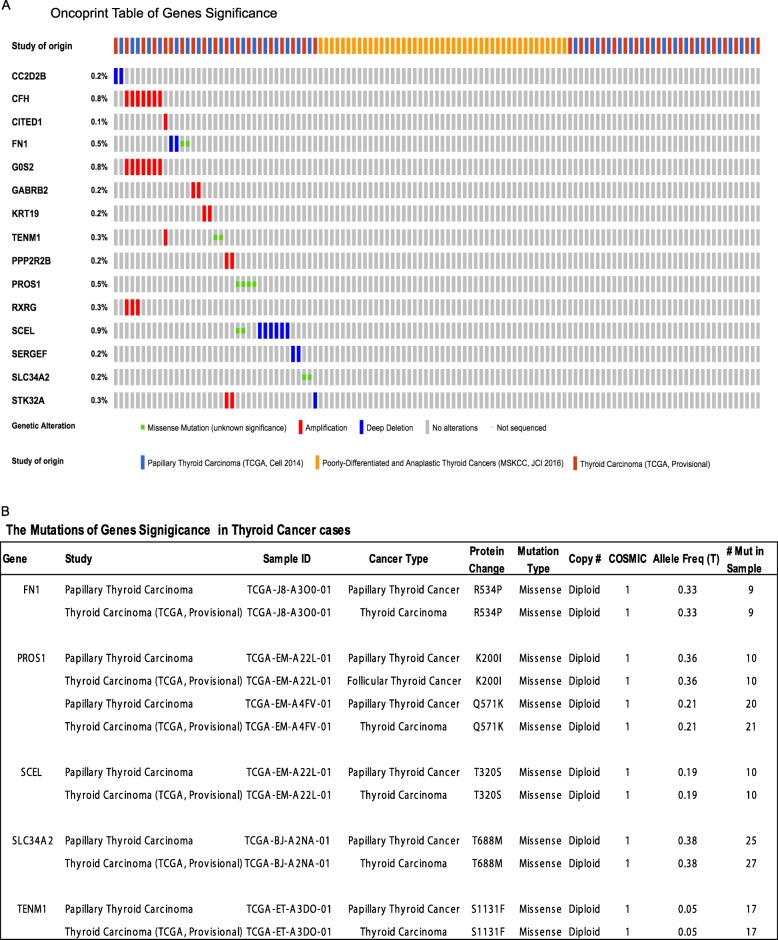
Validate Signature genes by Blast with independent cases via the cBioportal database. **a** Oncoprint table of significant signature genes. Through the cBioPortal Cancer Genomics database, the genes significance (GS) in blue module were explored multidimensional cancer genomics datasets in the context of clinical data and biologic pathways. The Oncoprint table summarizes genomic alterations in all queried genes across samples. Each row represents a gene, and each column represents a tumor sample. Red bars indicate gene amplifications, blue bars are homozygous deletions, and green squares are nonsynonymous mutations. **b** The summary Mutations table of query genes. The tabular view provides additional information about all mutations in each query genes

Moreover, we put these 24 genes into cBioPortal Cancer Genomics database for validation and inquired with 915 patients’ datasets in 3 independent studies as Papillary Thyroid Carcinoma (TCGA, Cell 2014), Thyroid Carcinoma (TCGA Provisional) and Poorly-Differentiated and Anaplastic Thyroid Cancer (MSKCC, JCI 2016). The exploring results indicated that 15 of 24 genes significance were altered in 37 (4.0%) of 915 queried cases/patients as listed in Oncoprint table (Fig. 4a). The matched genes are listed as *CC2D2B, CFH, CITED1, FN1, GOS2, GABRB2, KRT19, TENM1, PPP2R2B, PROS1, RXRG, SCEL, SERGEF, SLC34A2* and *STK32A.* The genetic alternation types included missense mutation, amplification and deep deletion (Fig. 4a). Sixteen genes that associated with detail information of copy-number alterations were identified, including the alternation type, altered samples number and percent of patient’s cases (Additional file Table S5). The exploring results contains 86 gene pairs with mutually exclusive alterations (none significant), and 167 gene pairs with co-occurrent alterations (non-significant) and 6 genes pairs with significant alternation (*P* value < 0.05). The 6 genes pairs are identified as *CFH & G0S2, CFH & RXRG, G0S2 & RXRG, PPP2R2B & STK32A, PROS1 & SCEL* and *CITED1* & *TENM1* (Additional file Table S6). It is summarized the detail of information about inquired genes genetic alternation (Additional file Table S7). The queried results discovered 5 genes significantly associated with missense mutation, *FN1* (R534P), *PROS1*((K200I), (Q571K)), *SCEL* (T320S), *SLC34A2*(T688M) and *TENM1* (S1131F), plus the key information about mutation type, protein change sites and mutation occurrence in patient’s case number (Fig. 4b). Furthermore, these 5 genes are also significantly higher expression in thyroid cancer cases (P value < 0.01) explored in TCGA database (Fig. 3c).

### Functional and gene ontology pathway enrichment analysis

The key functional pathway enrichment analysis was performed for the significant genes in Blue module. The significantly enriched pathways mainly concentrated in cell adhesion, extracellular matrix and low density lipoprotein metabolic, also included membrane-associated biological processes and cellular components (Table 1). There are 8 genes in Blue module and 4 clusters of significance enriched KEGG pathways identified by Genclip. The most significant top 2 cluster pathways are resorted to associated with Thyroid Cancer, small/non-small lung cancer or other cancers (Table 1, KEGG Pathway Analysis, cluster1 &2, Additional file Figure S6). The other 2 clusters pathways mainly involved in the cell adhesion, cell junction interaction & organization, platelet activation & degranulation, and leukocyte transendothelial migration (Table 1). The GO analysis identified 2 significant clusters functional associated with the responses to lipid, hormone, steroid hormone and organic cyclic compound (Table 1, GO Analysis, Additional file Figure S6A). According to previously research reports, 12 genes were involving in constructed a co-citation network. Through literature profiles analysis, the significant genes in blue module are mainly clustered in functions related to type 2 diabetes, cell adhesion, extracellular matrix and low density lipoprotein (Table 1, GO Analysis).

**Table 1:**
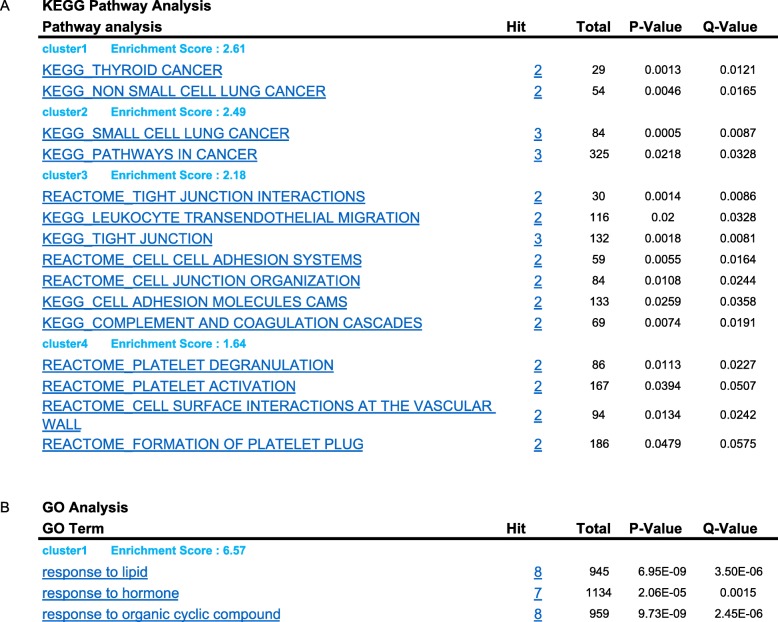
GO and KEGG pathway enrichment analysis of genes significance

## Discussion

In this study, to discover novel biomarkers to accelerate the precise clinical diagnostics for thyroid nodule cases with cytology indeterminate, we designed the whole project workflow, selected specific dataset (GSE34289) and applied the WGCNA approach to analyze the gene expression profiling of thyroid nodule that generated from FNA clinical samples (Fig. 1**)**. The gene expression profile contained 172 specific genes designed for promise diagnostic assessment [8], which are mainly involving in variously biological and cellular processes that related to energy metabolism, cell differentiation, and cellular development. and aimed to discover some novel biomarkers to accelerate the precise clinical diagnostics for thyroid cancer. It is representative to discover the signature genes significantly associated with thyroid nodules malignancy through gene expression profiling analysis of these cases. Furthermore, this dataset was generated from 49 national widely clinical sites, collected from 3789 patients and evaluated 4812 thyroid nodules samples (size > 1 cm) in United States and well characterized with higher standard. It obtained 577 cytological indeterminate aspirates and finally selected 265 indeterminate nodules for further analysis through blinded histopathological review [25]. In addition, this dataset also contained two groups of cases labelled as cytology-benign/histopathology-benign (44 cases) and cytology-malignant /histopathology-malignant (55) with validated cytopathology and histopathological features (Additional file Table S1). Based on this dataset characters, we utilized totally 364 samples and split into 4 groups in this study. The expression level of identified signature genes will be explored in cytology-benign/histopathology-benign and cytology-malignant/histopathology-malignant groups as negative and positive control. Moreover, as designed in the workflow, the discovered signature genes would be validated through another independently investigations of thyroid carcinomas patient’s cases in TCGA via GEIPA (Fig. 3c) and indicated these genes sensitiveness with statistical analysis.

Compared with other computational methodology, the WGCNA have unique merits, which could be robust and sensitive detection of the subset of genes co-expression as functional modules from the entire transcriptome and without pre-filtering to cause selective bias or losing useful information [20]. It was designed to discover the networks and genes associated with phenotypes of target by using unsupervised clustering and constructing gene module. The constructed gene co-expression module consists of a group of genes that maintain a consistent expression relationship and share a common biological regulation function that independent of a priori defined gene sets or pathways [31]. Previously, WGCNA has been successfully applied to biomarker discovery for cancer diagnostics, such as discovered microRNA expression network in prostate cancer [32] and identified ASPM as a potential biomarker in glioblastoma [33].

In our study, our results firstly demonstrated that it is reasonable to build on the co-expression networks with clinical traits (histopathology, age, gender) using Pearson correlation analysis (Additional file Figure S1). To discover the related modules to histopathological phenotype, we calculated the modules statistic significantly with the linear mixed effects model for testing the association of the node to the histological phenotype. We analyzed the gene expression profile data and identified three module eigengenes (ME), blue, Green and Turquoise module, are significantly associated with histological feature of malignant thyroid cancer (Fig. 2, Additional file Figure S4). Through the Eigengene dendrogram analysis, we discovered the most significantly hub, the blue module that contained 24 genes, related to histological feature (Fig. 2d&e).

To validate the WGCNA analysis results, we took two approaches to test signature genes positive expression and correlation. Firstly, we compared the signature genes expression level between sample group 2/3/4 and group one separately, which represent as test, negative and positive control. Gone through the results, we setup the fold change > 1.05 or fold change < 0.98 (plus *P* value < 0.001) as the cutoff for statistics significant standard. As results indicated, all 24 signature genes do not significant expression difference although some genes expression with lower or higher level in the double cytology/histopathology benign case group, which defined as negative control. While 22 signature genes were significantly higher expression (FC > 1.0, *P* value < 0.001), except *PPP2R2B* with lower expression (FC = 0.9227, *P* value = 0.0042) and *CC2D2B* with higher expression (FC = 1.0725, *P* value = 0.002), in the double cytology/histopathology malignant cases group, which works as positive control. These results indicated that 24 signature genes could significantly differentiate the malignancy and benign cases (positive rate = 91.67%, 22/24). For the cytology-Indeterminate/histopathology-malignant group, the 23 signature genes show significant higher expression (Fold change > 1.0, *P* value < 0.001) than benchmark (positive rate = 95.83%, 23/24), while only *PPP2R2B* with lower expression (*P* value = 0.0039) (Table 1). Furthermore, identified 5 potential biomarker genes were all significantly higher expression (FC > 1.15, *P* value < 0.0001) in both cytology-indeterminate/histopathology-malignant group and double cytology/histopathology malignant group (Fig. 3a&b). These results suggest these 5 genes have potential to be the biomarker candidate for differentiation the malignancy among the indeterminate cases. In the secondary approach, we explored these 24 genes through the cBioPortal Cancer Genomic database, which is containing many published cancer studies datasets from CCLE and TCGA [28], and verified through 3 independent thyroid cancer investigations that contained 915 patient’s datasets. The 16 genes were matched in 37 (4.0%) of 915 patient’s cases with genetic alternation of missense mutation, amplification, deep deletion and copy- number alterations, and listed as *CC2D2B, CFH, CITED1, FN1, GOS2, GABRB2, KRT19, TENM1, PPP2R2B, PROS1, RXRG, SCEL, SERGEF, SLC34A2* and *STK32A*. Some of these generic alternations were associated with papillary thyroid carcinoma metastasis to brain [34] and could be useful as histopathological biomarkers for papillary thyroid carcinoma [25, 35]. In addition, the queried results also discovered 5 genes with missense mutation significantly (*P* value < 0.01) associated thyroid cancer cases, listed as *FN1* (R534P), *PROS1* ((K200I), (Q571K)), *SCEL* (T320S), *SLC34A2*(T688M) and *TENM1* (S1131F) (Fig. 4b). We compared these genes expression level between groups, and our results indicated these genes could significantly differentiate the cases of benign and malignant among cytological indeterminate cases (Fig. 3a). In addition, 5 potential biomarkers significantly higher expression in malignant cases (P value < 0.0001, Fig. 3b). Furthermore, to validate these signature genes, we blast the 5 potential biomarkers through TGCA database with other independent clinical cases (about 849 cases, Fig. 3c). These 5 genes are also significantly higher expression in thyroid cancer cases (*P* value < 0.01) explored in GEPIA with thyroid cancer clinical cases data. These two validation approach made results more convincible.

Through the Genclip analysis, we found that these genes are mainly concentrate on the GO pathways that involving in physiological response of hormone and steroid hormone, and regulation of cell migration and adhesion, cell junction interaction, etc. (Table 1, Additional file Figure S6A**)**. The involving pathways are significantly concentrated in subgroups of thyroid cancer, non-small cell lung cancer and cancer, process, signaling, extracellar region, and transporter activity (Additional file Figure S6B, and Table 1). It indicates that these functions may be associated with metabolism and accelerated growth and development of obesity individuals. Notably, the results of GO enrichment analysis also provide more significant pathways with biological annotations (Table 1).

Checked with published literature, these 5 genes were reported associated with the transform progress of multiple carcinomas. Fibronectin 1 (*FN1*) is a glycoprotein existing with soluble dimer or multimeric form in different conditions. *FN1* is involved in multiple cell adhesion and migration processes, including embryogenesis, wound healing, blood coagulation, host defense, and found with higher expression in metastasis [36]. It was reported that *FN1* is over expression in the Papillary Thyroid Carcinoma [37] and listed as potential biomarker for diagnostics. *PROS1*(Protein S 1) is a vitamin K-dependent plasma protein that works as a cofactor of the anticoagulant protease. It could activate protein C (APC) and inhibit blood coagulation [38]. The genetic mutation of this gene will result in autosomal dominant hereditary thrombophilia [39] and malignant glioma [40]. The *PROS1* and *FN1* others 12 genes alternations were identified as important diagnostic biomarkers for thyroid cancer through the meta-analysis the gene expression profiling of clinical thyroid nodules [41]. *SCEL* (Sciellin) is the precursor to the cornified envelope of terminally differentiated keratinocytes. *SCEL* is overexpressed in the papillary thyroid carcinoma and worked as key regulator in mesenchymal-to-epithelial transition and dynamically regulated through the metastasis process [36]. *SCEL* was high expression in thyroid tumor tissue and significantly associated with I-131 [42]. *TENM1* (Teneurin transmembrane protein 1) is involving in pattern formation and morphogenesis [43]. *TENM1* was overexpression in thyroid cancer and associated with thyroidal invasion [44] and identified as potential marker of papillary thyroid carcinoma progress [36, 45]. *SLC34A2* (solute carrier family 34 member 2) is a member of the *SLC34* solute carrier protein family and coded for pH-sensitive sodium-dependent phosphate transporter (NaPi2b), which is a multi-transmembrane [46] and The physiological function of *SLC34A2* is transcellular inorganic phosphate absorption and maintenance of phosphate homeostasis [47] and cell differentiation. *SLC34A2* is overexpressed in multiple cancer types, including lung, ovarian, and thyroid cancers [48] and identified as potential therapeutic target for non-small cell lung and Ovarian cancer [48]. Combined with these independent research results, it is strong suggest that *FN1* (R534P), *PROS1* ((K200I), (Q571K)), *SCEL* (T320S), *SLC34A2* (T688M) and *TENM1* (S1131F) are potential novel biomarker candidates significantly associated with thyroid carcinomas and could differentiate the malignant thyroid nodule among cytological indeterminate cases. As mentioned previously, the 7-gene MT biomarkers panel was broadly recommended to evaluate the residual cytological indeterminate thyroid nodules and estimate with high specificity (~ about 90%) [14–16]. However, the sensitivity of 7-gene MT biomarkers panel testing showed huge variation (from 44 to 100%) in clinical practice [6, 17]. It suggests that there are some unknown biomarkers existing in these indeterminate cases. It is possible that our identified 5 novel biomarkers genes could contribute to enhance specificity of previously 7-gene MT biomarkers panel. The combined application of these two panel biomarkers would get more promise and precise clinical diagnostic results for nodules malignancy in some cases population.

There are some limitations and several novelties in our study. The FNA yield cytology indeterminate cases are including subtype of follicular lesion, follicular neoplasm and suspicious or malignancy. For the first limitation from this dataset, lacking of these cases histopathological information about thyroid cancer subtype sorting, such as follicular adenoma (FA), follicular carcinoma (FC) and papillary thyroid carcinoma (PTC), we could not track these potential biomarkers back to original patient’s pathological status and dig deeper insight. Secondly, the gene expression profile was limited to 172 genes for promise diagnostic assessment [8]. It will cause to miss other genes significantly associated with malignant thyroid carcinomas by this pre-filter selection. Thirdly, due to the bioinformatics analysis nature, the discovered specific GO pathways and KEGG pathways were referred from previously literatures and did not be further investigated. Although we explored these significant genes associated with histological feature through TCGA database via cBioPortal and compared with the other two groups case in the same dataset, these potential biomarkers will be required to verify with storing patient’s cases according to subtype of thyroid cancer by immunohistochemistry (IHC) or other genetic detection method, like qPCR or sequencing in coming research work. Therefore, more number and sorting subtype patient’s cases are mandatory to verify these potential biomarkers for thyroid cancer precise diagnostics in the future cohort study. On the other side, our study has several novelties. Firstly, we applied reverse strategy by using WGCNA approach to discover the genes significantly associated with malignant histopathological feature in clinical FNA samples with cytological indeterminate feature. In parallels, compared these signature genes expression level among histopathological benign and malignant groups, the results indicated that signature genes have significant positive overexpression in Malignant groups and negative overexpression in benign group (Table 1). Secondly, we inquired the key functional & GO pathways that genes significance in module involving in the progress of thyroid carcinomas by Genclip enrichment analysis (Table 1). The results will be a clause for the next step research. Thirdly, exploring through TCGA database, we discovered 5 novel potential biomarkers to differentiate the malignant and benign thyroid nodules, which were identified as potential biomarkers of malignant thyroid cancer in previously independent researches. Furthermore, these 5 genes were validated with significant higher expression level in the TCGA thyroid cancer cases (Fig. 3c). These results are partially as evidences to support our results and research strategy.

## Conclusions

Our study identified five novel signature genes with missense mutation, *FN1* (R534P), *PROS1*((K200I), (Q571K)), *SCEL* (T320S), *SLC34A2*(T688M) and *TENM1* (S1131F) that highlighted as potential biomarkers to rule out nodules malignancy. These novel results provide new insight and strategy to identify these potential biomarkers and differentiate malignant histopathological thyroid nodules with cytological Indeterminate. The clinical validation and application of these prognostic biomarkers will facilitate the precise diagnostics and help to enhance the healthcare efficiency for thyroid cancer patients.

## Supplementary information

**Additional file 1: Figure S1.** Sample dendrogram and Clinic Feature traits heatmap. Clustering dendrogram of samples based on their Euclidean distance. The clinical feature traits were histopathology, gender and age. The white color means a low value, red means a high value, and grey represents a missing entry.

**Additional file 2: Figure S2.** Analysis of network topology for various soft-thresholding powers. In panel (A), the scale-free topology model fit index (signed R^2^, y-axis) shows as a function of the soft-thresholding power (x-axis). In panel (B), the mean connectivity (ki, y-axis) displays as a function of the soft-thresholding power (x-axis) under different weighting coefficients. The connectivity ki of node i equals the number of its direct connections to other nodes. P(k) indicates the frequency distribution of the connectivity. The higher the coefficient, the closer the network is to the distribution of the scale free network.

**Additional file 3: Figure S3.** Heatmap plot of genes network. The heatmap represents the Topological Overlap Matrix (TOM) among all Genes used for analysis. Light color represents low overlap and progressively darker red color represents higher overlap. Blocks of darker colors along the diagonal are the modules. The gene dendrogram and module assignment are also shown along the left side and the top.

**Additional file 4: Figure S4.** Clustering dendrogram of Genes, with dissimilarity based on topological overlap. Different colors index different modules. Six modules are identified. Grey bars represent Genes that do not belong to any other modules and are not co-expressed.

**Additional file 5: Figure S5.** The scatterplots of Gene Significance (GS) for histology vs. Module Membership (MM) in the all modules (A~E). There is a highly significant correlation between GS and MM in this module, implying that the most important (central) elements of blue module also tend to be highly correlated with thyroid nodule histology trait.

**Additional file 6: Figure S6.** GO and pathway analysis of Genes Significant (GS). Clustering analysis of the biological functions of 22 genes in previous studies for GO (A) and Pathway (B) generated by the GenClip software. In the heatmap, the black color represents that the biological function of the corresponding gene-term association has not been reported yet. While light green color means that the corresponding gene-term association positively has been reported. The color scale bar for proportion of genes associated were labelled.

**Additional file 7: Table S1.** The samples information and case group sorting.

**Additional file 8: Table S2.** The pick soft threshold for Module.

**Additional file 9: Table S3.** List of the 29 Genes in the grey module.

**Additional file 10: Table S4.** List of the 24 significant genes in the blue module.

**Additional file 11: Table S5.** The Copy-number Alterations of significant genes.

**Additional file 12: Table S6.** The Mutual Exclusivity tab of significant gene pairs. The genes pairs alternated in Thyroid cancer are mutual exclusivity. The tab provides summary statistics significant on mutual exclusivity and co-occurrence of genomic alterations in each pair of query genes. The mutual exclusivity is significant for the other two gene pairs (*P* < 0.05). The *P* values are determined by a Fisher’s exact test with the null hypothesis that the frequency of occurrence of a pair of alterations in two genes is proportional to their uncorrelated occurrence in each gene.

**Additional file 13: Table S7.** The Genetic Alterations type of query genes.

## Data Availability

The original data used in this study was mentioned in the section “Gene Expression Data Source” [GSE34289]. The secondary datasets used or generated by analysis in this study are available from online supplementary.

## Abbreviations

7-gene MT: 7-gene molecular panel
CITED1: Cbp/p300 Interacting transactivator with Glu/Asp rich Carboxy-terminal domain 1
FN1: Fibronectin 1
FNA: Fine-needle aspiration
GO: Gene ontology
GS: Gene significance
ME: Module eigengene
MM: Module membership
MS: Module significance
PCA: Principal component analysis
PROS1: Protein S
SCEL: Sciellin
SLC34A2: Solute carrier family 34 member 2
TCGA: The Cancer Genome Atlas
TENM1: Teneurin transmembrane protein 1
TOM: Topological Overlap Matrix
WGCNA: Weighted gene co-expression network analysis

## References

[1] Chen W, Zheng R, Baade PD, Zhang S, Zeng H, Bray F, Jemal A, Yu XQ, He J (2016). Cancer statistics in China, 2015. CA Cancer J Clin.

[2] Chen AY, Jemal A, Ward EM (2009). Increasing incidence of differentiated thyroid cancer in the United States, 1988-2005. Cancer.

[3] Treglia G, Aktolun C, Chiti A, Frangos S, Giovanella L, Hoffmann M, Iakovou I, Mihailovic J, Krause BJ, Langsteger W (2016). The 2015 revised American Thyroid Association guidelines for the management of medullary thyroid carcinoma: the “evidence-based” refusal to endorse them by EANM due to the “not evidence-based” marginalization of the role of nuclear medicine. Eur J Nucl Med Mol Imaging.

[4] Bak M, Peter I, Nyari T, Simon P, Ujlaky M, Boer A, Kasler M (2015). On-site fine-needle aspiration cytology of thyroid nodules. Quality assurance of the Bethesda system for reporting thyroid cytopathology (2008). Orv Hetil.

[5] Mallick UK, American Thyroid A (2010). The revised American Thyroid Association management guidelines 2009 for patients with differentiated thyroid cancer: an evidence-based risk-adapted approach. Clin Oncol (R Coll Radiol).

[6] Nikiforov YE, Ohori NP, Hodak SP, Carty SE, LeBeau SO, Ferris RL, Yip L, Seethala RR, Tublin ME, Stang MT (2011). Impact of mutational testing on the diagnosis and management of patients with cytologically indeterminate thyroid nodules: a prospective analysis of 1056 FNA samples. J Clin Endocrinol Metab.

[7] Wang CC, Friedman L, Kennedy GC, Wang H, Kebebew E, Steward DL, Zeiger MA, Westra WH, Wang Y, Khanafshar E (2011). A large multicenter correlation study of thyroid nodule cytopathology and histopathology. Thyroid.

[8] Chudova D, Wilde JI, Wang ET, Wang H, Rabbee N, Egidio CM, Reynolds J, Tom E, Pagan M, Rigl CT (2010). Molecular classification of thyroid nodules using high-dimensionality genomic data. J Clin Endocrinol Metab.

[9] Pagedar NA, Chen DH, Wasman JK, Savvides P, Schluchter MD, Wilhelm SM, Lavertu P (2008). Molecular classification of thyroid nodules by cytology. Laryngoscope.

[10] Nam SY, Han BK, Ko EY, Kang SS, Hahn SY, Hwang JY, Nam MY, Kim JW, Chung JH, Oh YL (2010). BRAF V600E mutation analysis of thyroid nodules needle aspirates in relation to their ultrasongraphic classification: a potential guide for selection of samples for molecular analysis. Thyroid.

[11] Cancer Genome Atlas Research N (2014). Integrated genomic characterization of papillary thyroid carcinoma. Cell.

[12] Grogan RH, Mitmaker EJ, Clark OH (2010). The evolution of biomarkers in thyroid cancer-from mass screening to a personalized biosignature. Cancers (Basel).

[13] Yip L, Ferris RL (2014). Clinical application of molecular testing of fine-needle aspiration specimens in thyroid nodules. Otolaryngol Clin N Am.

[14] Giordano TJ, Beaudenon-Huibregtse S, Shinde R, Langfield L, Vinco M, Laosinchai-Wolf W, Labourier E (2014). Molecular testing for oncogenic gene mutations in thyroid lesions: a case-control validation study in 413 postsurgical specimens. Hum Pathol.

[15] Beaudenon-Huibregtse S, Alexander EK, Guttler RB, Hershman JM, Babu V, Blevins TC, Moore P, Andruss B, Labourier E (2014). Centralized molecular testing for oncogenic gene mutations complements the local cytopathologic diagnosis of thyroid nodules. Thyroid.

[16] Nayar R, Ivanovic M (2009). The indeterminate thyroid fine-needle aspiration: experience from an academic center using terminology similar to that proposed in the 2007 national cancer institute thyroid fine needle aspiration state of the science conference. Cancer.

[17] Nikiforov YE, Steward DL, Robinson-Smith TM, Haugen BR, Klopper JP, Zhu Z, Fagin JA, Falciglia M, Weber K, Nikiforova MN (2009). Molecular testing for mutations in improving the fine-needle aspiration diagnosis of thyroid nodules. J Clin Endocrinol Metab.

[18] Haugen BR, Alexander EK, Bible KC, Doherty GM, Mandel SJ, Nikiforov YE, Pacini F, Randolph GW, Sawka AM, Schlumberger M (2016). 2015 American Thyroid Association management guidelines for adult patients with thyroid nodules and differentiated thyroid cancer: the American Thyroid Association guidelines task force on thyroid nodules and differentiated thyroid cancer. Thyroid.

[19] Haugen BR (2017). 2015 American Thyroid Association management guidelines for adult patients with thyroid nodules and differentiated thyroid cancer: what is new and what has changed?. Cancer.

[20] Zhang B, Horvath S (2005). A general framework for weighted gene co-expression network analysis. Stat Appl Genet Mol Biol.

[21] Zhang J, Baddoo M, Han C, Strong MJ, Cvitanovic J, Moroz K, Dash S, Flemington EK, Wu T (2016). Gene network analysis reveals a novel 22-gene signature of carbon metabolism in hepatocellular carcinoma. Oncotarget.

[22] Guo Y, Xing Y (2016). Weighted gene co-expression network analysis of pneumocytes under exposure to a carcinogenic dose of chloroprene. Life Sci.

[23] Zhu XL, Ai ZH, Wang J, Xu YL, Teng YC (2012). Weighted gene co-expression network analysis in identification of endometrial cancer prognosis markers. Asian Pac J Cancer Prev.

[24] Shi K, Bing ZT, Cao GQ, Guo L, Cao YN, Jiang HO, Zhang MX (2015). Identify the signature genes for diagnose of uveal melanoma by weight gene co-expression network analysis. Int J Ophthalmol.

[25] Alexander EK, Kennedy GC, Baloch ZW, Cibas ES, Chudova D, Diggans J, Friedman L, Kloos RT, LiVolsi VA, Mandel SJ (2012). Preoperative diagnosis of benign thyroid nodules with indeterminate cytology. N Engl J Med.

[26] Langfelder P, Horvath S (2008). WGCNA: an R package for weighted correlation network analysis. BMC Bioinformatics.

[27] Li A, Horvath S (2007). Network neighborhood analysis with the multi-node topological overlap measure. Bioinformatics.

[28] Cerami E, Gao J, Dogrusoz U, Gross BE, Sumer SO, Aksoy BA, Jacobsen A, Byrne CJ, Heuer ML, Larsson E (2012). The cBio cancer genomics portal: an open platform for exploring multidimensional cancer genomics data. Cancer Discov.

[29] Gao J, Aksoy BA, Dogrusoz U, Dresdner G, Gross B, Sumer SO, Sun Y, Jacobsen A, Sinha R, Larsson E (2013). Integrative analysis of complex cancer genomics and clinical profiles using the cBioPortal. Sci Signal.

[30] Huang ZX, Tian HY, Hu ZF, Zhou YB, Zhao J, Yao KT (2008). GenCLiP: a software program for clustering gene lists by literature profiling and constructing gene co-occurrence networks related to custom keywords. BMC Bioinformatics.

[31] Stuart JM, Segal E, Koller D, Kim SK (2003). A gene-coexpression network for global discovery of conserved genetic modules. Science.

[32] Wang L, Tang H, Thayanithy V, Subramanian S, Oberg AL, Cunningham JM, Cerhan JR, Steer CJ, Thibodeau SN (2009). Gene networks and microRNAs implicated in aggressive prostate cancer. Cancer Res.

[33] Horvath S, Zhang B, Carlson M, Lu KV, Zhu S, Felciano RM, Laurance MF, Zhao W, Qi S, Chen Z (2006). Analysis of oncogenic signaling networks in glioblastoma identifies ASPM as a molecular target. Proc Natl Acad Sci U S A.

[34] Schulten HJ, Hussein D, Al-Adwani F, Karim S, Al-Maghrabi J, Al-Sharif M, Jamal A, Bakhashab S, Weaver J, Al-Ghamdi F (2016). Microarray expression profiling identifies genes, including cytokines, and biofunctions, as diapedesis, associated with a brain metastasis from a papillary thyroid carcinoma. Am J Cancer Res.

[35] Nakamura N, Erickson LA, Jin L, Kajita S, Zhang H, Qian X, Rumilla K, Lloyd RV (2006). Immunohistochemical separation of follicular variant of papillary thyroid carcinoma from follicular adenoma. Endocr Pathol.

[36] Huang Y, Prasad M, Lemon WJ, Hampel H, Wright FA, Kornacker K, LiVolsi V, Frankel W, Kloos RT, Eng C (2001). Gene expression in papillary thyroid carcinoma reveals highly consistent profiles. Proc Natl Acad Sci U S A.

[37] da Silveira Mitteldorf CA, de Sousa-Canavez JM, Leite KR, Massumoto C, Camara-Lopes LH (2011). FN1, GALE, MET, and QPCT overexpression in papillary thyroid carcinoma: molecular analysis using frozen tissue and routine fine-needle aspiration biopsy samples. Diagn Cytopathol.

[38] Dahlback B, Stenflo J (1981). High molecular weight complex in human plasma between vitamin K-dependent protein S and complement component C4b-binding protein. Proc Natl Acad Sci U S A.

[39] Taniguchi F, Morishita E, Sekiya A, Nomoto H, Katsu S, Kaneko S, Asakura H, Ohtake S (2017). Gene analysis of six cases of congenital protein S deficiency and functional analysis of protein S mutations (A139V, C449F, R451Q, C475F, A525V and D599TfsTer13). Thromb Res.

[40] Milinkovic V, Bankovic J, Rakic M, Stankovic T, Skender-Gazibara M, Ruzdijic S, Tanic N (2013). Identification of novel genetic alterations in samples of malignant glioma patients. PLoS One.

[41] Griffith OL, Melck A, Jones SJ, Wiseman SM (2006). Meta-analysis and meta-review of thyroid cancer gene expression profiling studies identifies important diagnostic biomarkers. J Clin Oncol.

[42] Abend M, Pfeiffer RM, Ruf C, Hatch M, Bogdanova TI, Tronko MD, Hartmann J, Meineke V, Mabuchi K, Brenner AV (2013). Iodine-131 dose-dependent gene expression: alterations in both normal and tumour thyroid tissues of post-Chernobyl thyroid cancers. Br J Cancer.

[43] Tucker RP, Chiquet-Ehrismann R (2006). Teneurins: a conserved family of transmembrane proteins involved in intercellular signaling during development. Dev Biol.

[44] Nikolova DN, Zembutsu H, Sechanov T, Vidinov K, Kee LS, Ivanova R, Becheva E, Kocova M, Toncheva D, Nakamura Y (2008). Genome-wide gene expression profiles of thyroid carcinoma: identification of molecular targets for treatment of thyroid carcinoma. Oncol Rep.

[45] Cheng SP, Chen MJ, Chien MN, Lin CH, Lee JJ, Liu CL (2017). Overexpression of teneurin transmembrane protein 1 is a potential marker of disease progression in papillary thyroid carcinoma. Clin Exp Med.

[46] Xu H, Bai L, Collins JF, Ghishan FK (1999). Molecular cloning, functional characterization, tissue distribution, and chromosomal localization of a human, small intestinal sodium-phosphate (Na+-Pi) transporter (SLC34A2). Genomics.

[47] Virkki LV, Biber J, Murer H, Forster IC (2007). Phosphate transporters: a tale of two solute carrier families. Am J Physiol Renal Physiol.

[48] Lin K, Rubinfeld B, Zhang C, Firestein R, Harstad E, Roth L, Tsai SP, Schutten M, Xu K, Hristopoulos M (2015). Preclinical development of an anti-NaPi2b (SLC34A2) antibody-drug conjugate as a therapeutic for non-small cell lung and ovarian cancers. Clin Cancer Res.

