# Supplementary material for: Identification of potential novel biomarkers to differentiate malignant thyroid nodules with cytological indeterminate
Source: BMC Cancer. 2020 Mar 12;20:199. doi: 10.1186/s12885-020-6676-z (PMC7066786; doi:10.1186/s12885-020-6676-z)
Supplement: Supplementary file 1 — Additional file 1: Figure S1. Sample dendrogram and Clinic Feature traits heatmap. Clustering dendrogram of samples based on their Euclidean distance. The clinical feature traits were histopathology, gender and age. The white color means a low value, red means a high value, and grey represents a missing entry. [file 12885_2020_6676_MOESM1_ESM.pdf]

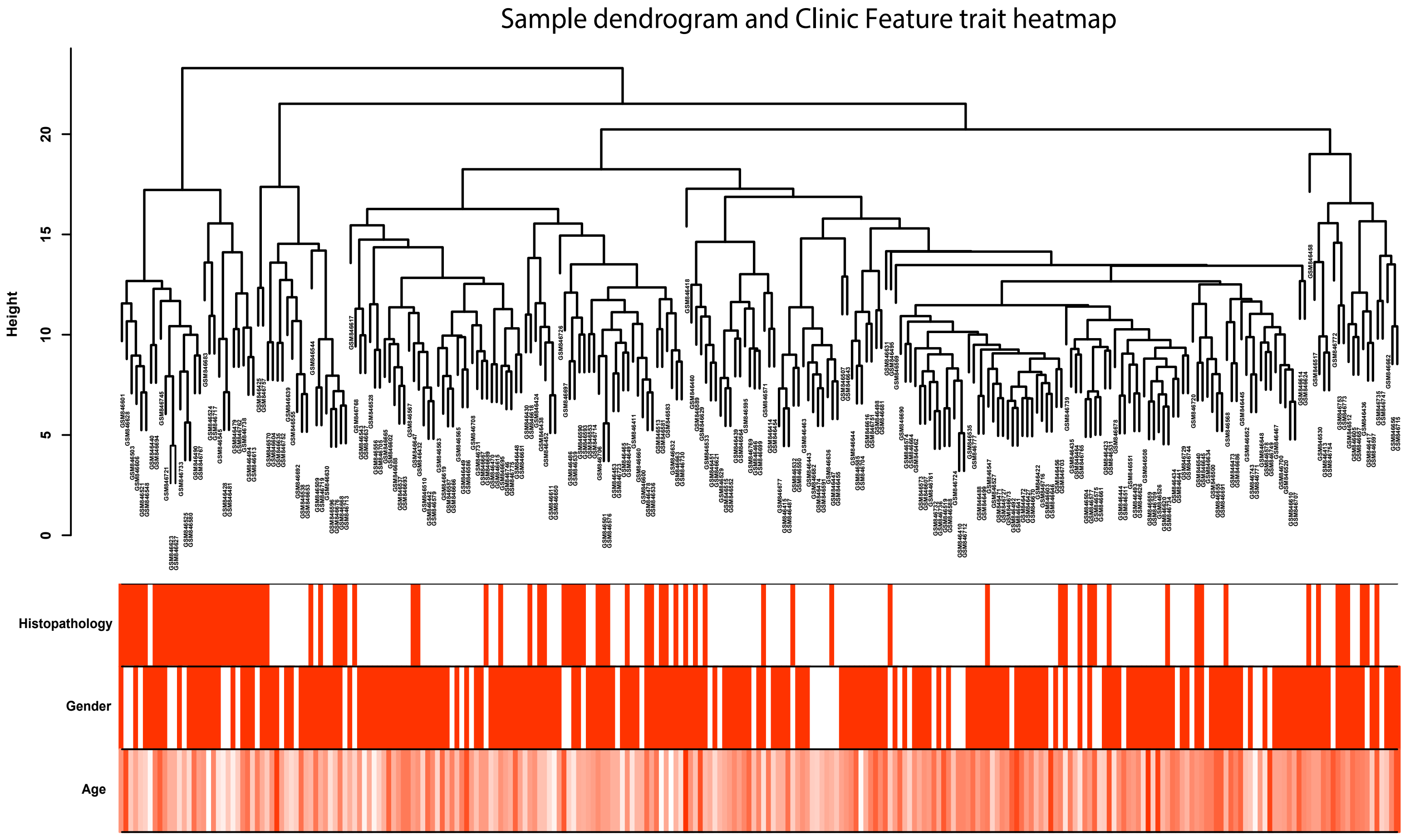

Supporting Figure 1. Sample dendrogram and clinic feature traits heatmap. Clustering dendrogram of samples based on their euclidean distance. The clinical feature traits were histopathology, gender and age. The white color means a low value, red means a high value, and grey represents a missing entry.
