# Supplementary material for: Identification of potential novel biomarkers to differentiate malignant thyroid nodules with cytological indeterminate
Source: BMC Cancer. 2020 Mar 12;20:199. doi: 10.1186/s12885-020-6676-z (PMC7066786; doi:10.1186/s12885-020-6676-z)
Supplement: Supplementary file 2 — Additional file 2: Figure S2. Analysis of network topology for various soft-thresholding powers. In panel (A), the scale-free topology model fit index (signed R2, y-axis) shows as a function of the soft-thresholding power (x-axis). In panel (B), the mean connectivity (ki, y-axis) displays as a function of the soft-thresholding power (x-axis) under different weighting coefficients. The connectivity ki of node i equals the number of its direct connections to other nodes. P(k) indicates the frequency distribution of the connectivity. The higher the coefficient, the closer the network is to the distribution of the scale free network. [file 12885_2020_6676_MOESM2_ESM.pdf]

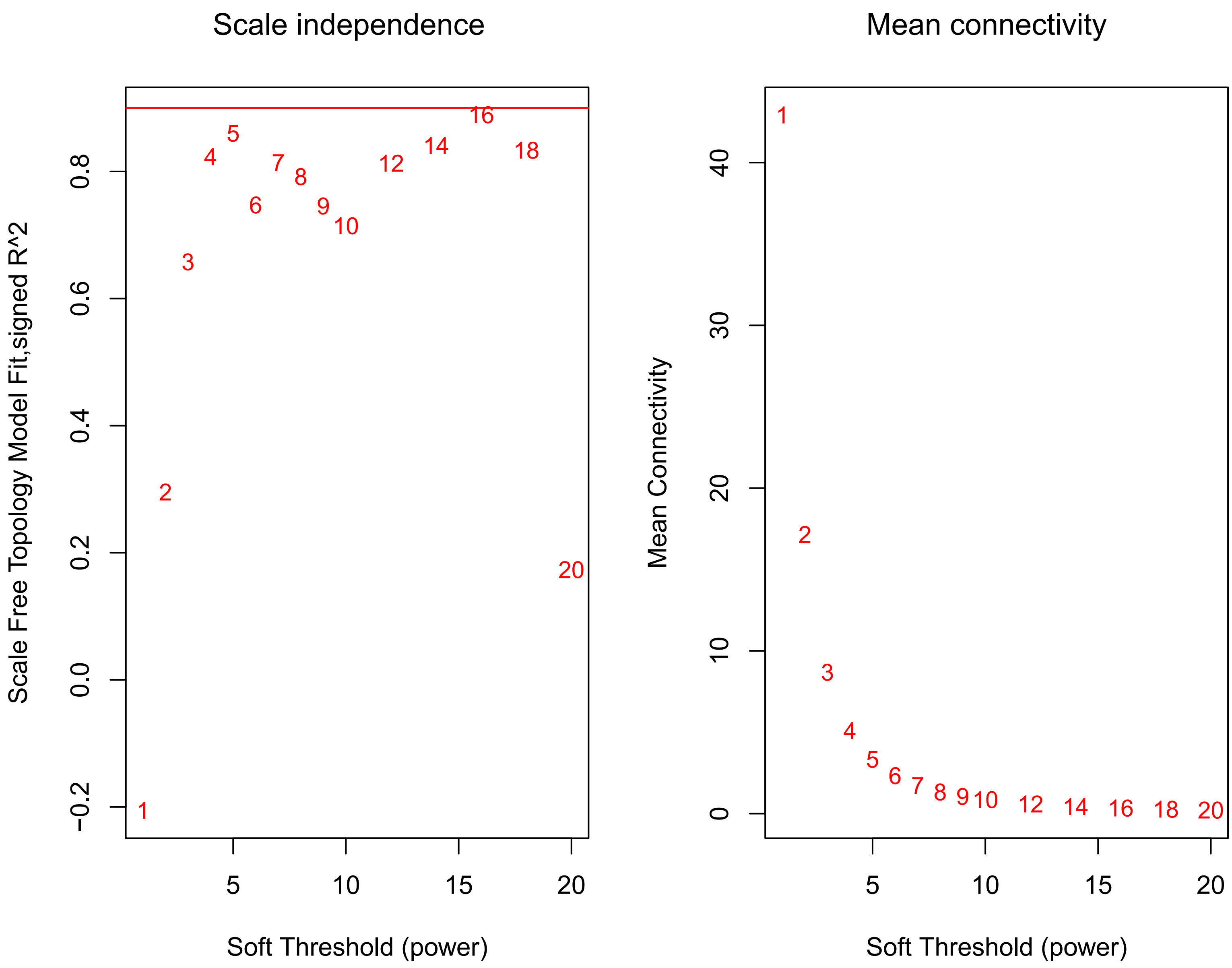

Supporting Figure 2. Analysis of network topology for various soft-thresholding powers. In Panel (A), the scale-free topology model fit index (Signed R2, y-axis) shows as a function of the soft-thresholding power (x-axis). In Panel (B), the mean connectivity (ki, y-axis) displays as a function of the soft-thresholding power (x-axis) under different weighting coefficients. The connectivity ki of node i equals the number of its direct connections to other nodes. P(k) indicates the frequency distribution of the connectivity. The higher the coefficient, the closer the network is to the distribution of the scale free network.
