# Supplementary material for: Identification of potential novel biomarkers to differentiate malignant thyroid nodules with cytological indeterminate
Source: BMC Cancer. 2020 Mar 12;20:199. doi: 10.1186/s12885-020-6676-z (PMC7066786; doi:10.1186/s12885-020-6676-z)
Supplement: Supplementary file 3 — Additional file 3: Figure S3. Heatmap plot of genes network. The heatmap represents the Topological Overlap Matrix (TOM) among all Genes used for analysis. Light color represents low overlap and progressively darker red color represents higher overlap. Blocks of darker colors along the diagonal are the modules. The gene dendrogram and module assignment are also shown along the left side and the top. [file 12885_2020_6676_MOESM3_ESM.pdf]

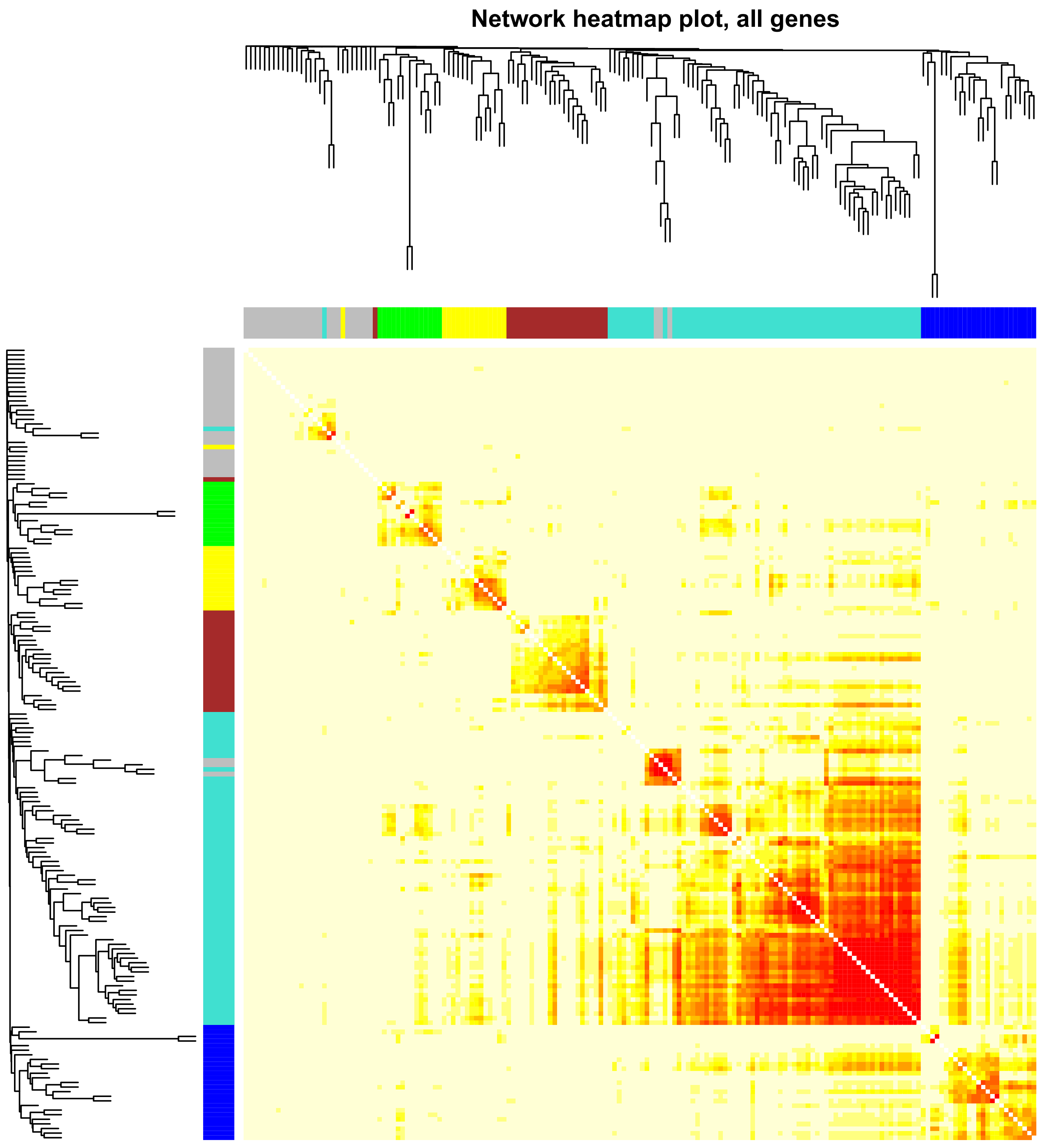

Supporting Figure 3. Heatmap plot of genes network. The heatmap represents the Topological Overlap Matrix (TOM) among all Genes used for analysis. Light color represents low overlap and progressively darker red color represents higher overlap. Blocks of darker colors along the diagonal are the modules. The gene dendrogram and module assignment are also shown along the left side and the top.
