# Supplementary material for: Identification of potential novel biomarkers to differentiate malignant thyroid nodules with cytological indeterminate
Source: BMC Cancer. 2020 Mar 12;20:199. doi: 10.1186/s12885-020-6676-z (PMC7066786; doi:10.1186/s12885-020-6676-z)
Supplement: Supplementary file 4 — Additional file 4: Figure S4. Clustering dendrogram of Genes, with dissimilarity based on topological overlap. Different colors index different modules. Six modules are identified. Grey bars represent Genes that do not belong to any other modules and are not co-expressed. [file 12885_2020_6676_MOESM4_ESM.pdf]

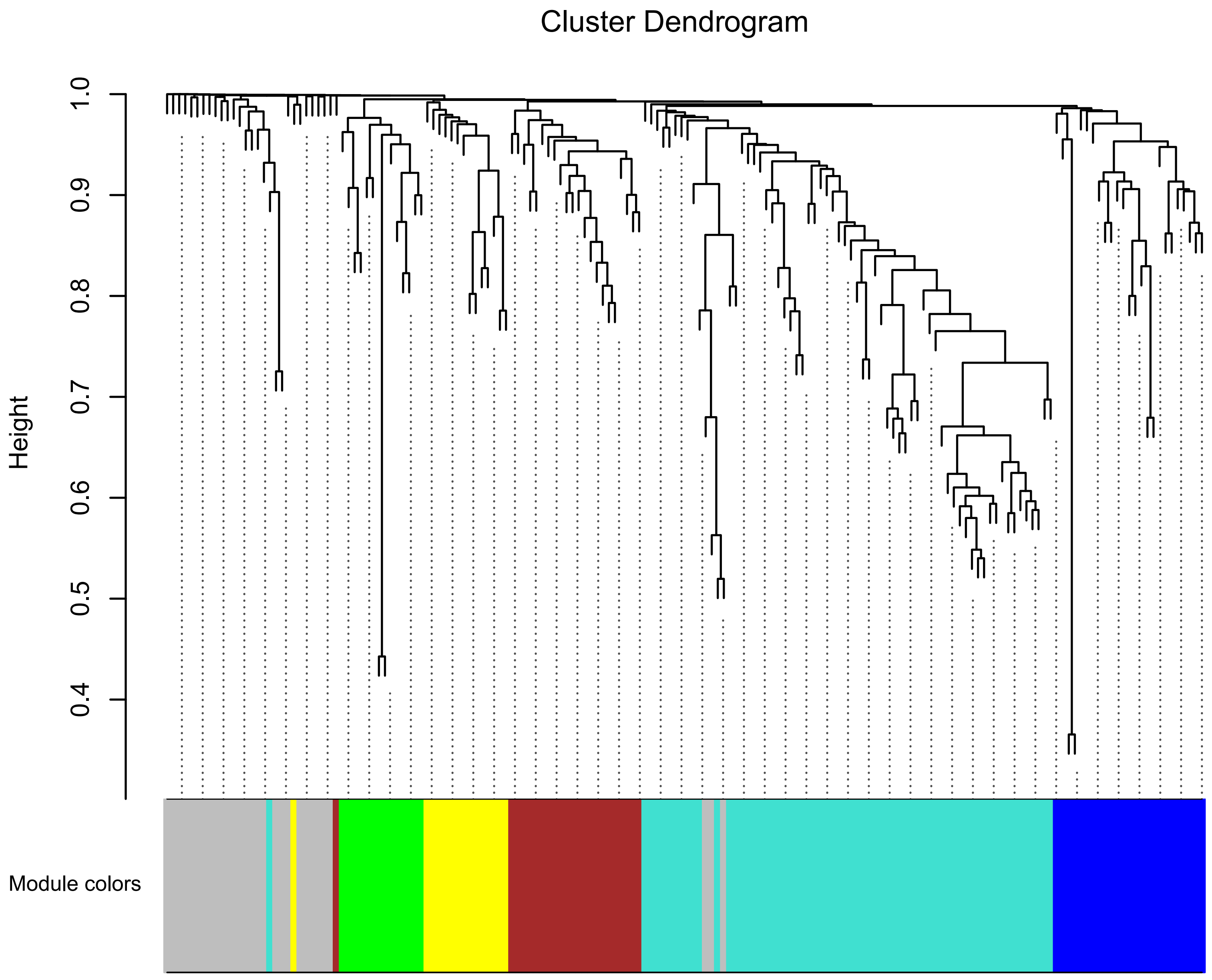

Supporting Figure 4. .Clustering dendrogram of Genes, with dissimilarity based on topological overlap. Different colors index different modules. Six modules are identified. Grey bars represent genes that do not belong to any other modules and are not co-expressed.
