# Supplementary material for: Identification of potential novel biomarkers to differentiate malignant thyroid nodules with cytological indeterminate
Source: BMC Cancer. 2020 Mar 12;20:199. doi: 10.1186/s12885-020-6676-z (PMC7066786; doi:10.1186/s12885-020-6676-z)
Supplement: Supplementary file 5 — Additional file 5: Figure S5. The scatterplots of Gene Significance (GS) for histology vs. Module Membership (MM) in the all modules (A~E). There is a highly significant correlation between GS and MM in this module, implying that the most important (central) elements of blue module also tend to be highly correlated with thyroid nodule histology trait. [file 12885_2020_6676_MOESM5_ESM.pdf]

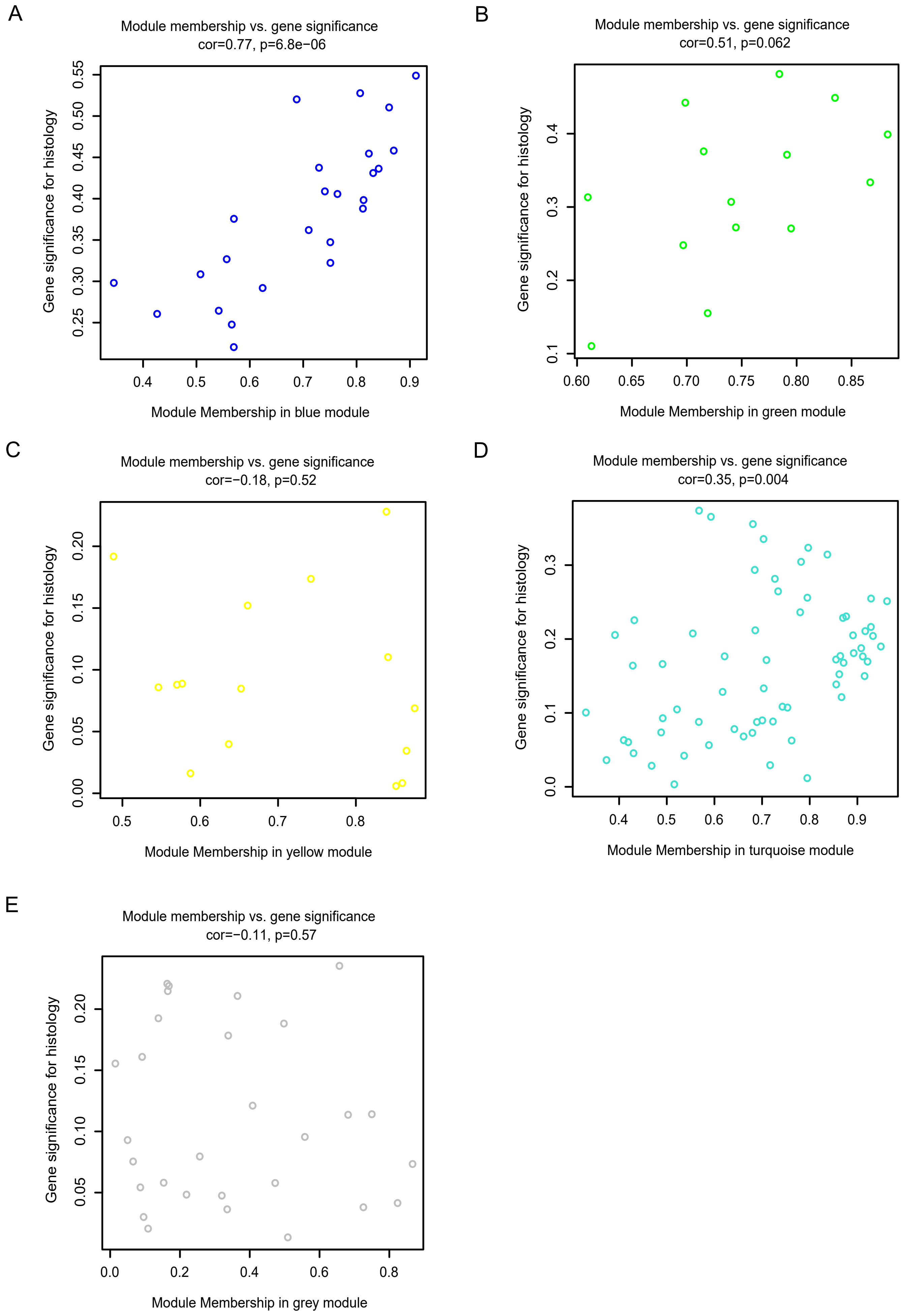

Supporting Figure 5. The scatterplots of Gene Significance (GS) for histology vs. Module Membership (MM) in the all modules (A-E). There is a highly significant correlation between GS and MM in this module, implying that the most important (central) elements of blue module also tend to be highly correlated with thyroid nodule histology trait.
