# Supplementary material for: Identification of potential novel biomarkers to differentiate malignant thyroid nodules with cytological indeterminate
Source: BMC Cancer. 2020 Mar 12;20:199. doi: 10.1186/s12885-020-6676-z (PMC7066786; doi:10.1186/s12885-020-6676-z)
Supplement: Supplementary file 6 — Additional file 6: Figure S6. GO and pathway analysis of Genes Significant (GS). Clustering analysis of the biological functions of 22 genes in previous studies for GO (A) and Pathway (B) generated by the GenClip software. In the heatmap, the black color represents that the biological function of the corresponding gene-term association has not been reported yet. While light green color means that the corresponding gene-term association positively has been reported. The color scale bar for proportion of genes associated were labelled. [file 12885_2020_6676_MOESM6_ESM.pdf]

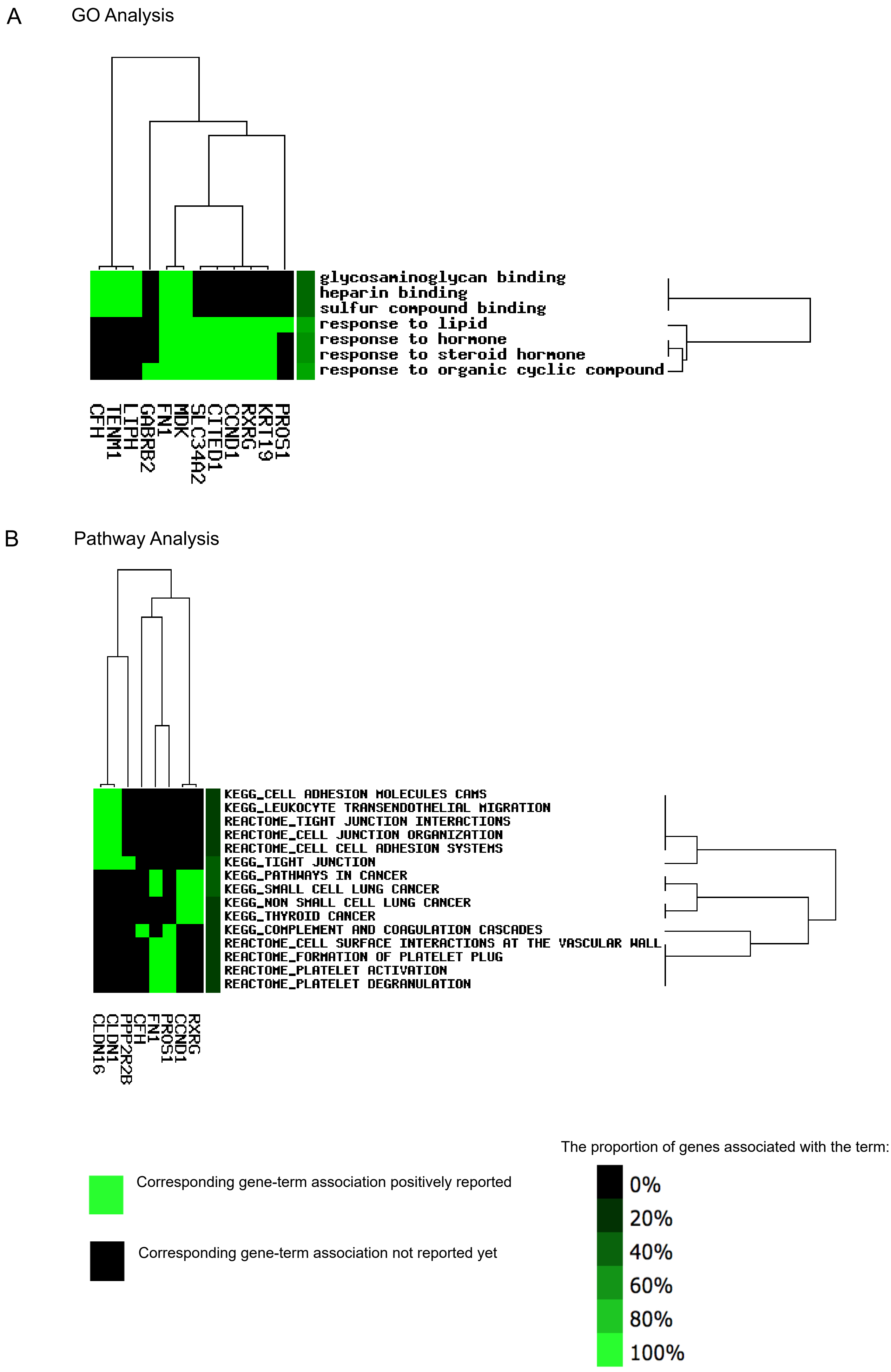

Supporting Figure 6. GO and pathway analysis of Genes Significant (GS). Clustering analysis of the biological functions of 22 genes in previous studies for GO (A) and Pathway (B) generated by the GenClip software. In the heatmap, the black color represents that the biological function of the corresponding gene-term association has not been reported yet. While light green color means that the corresponding gene-term association positively has been reported. The color scale bar for proportion of genes associated were labelled.
