# Supplementary material for: Identification of potential novel biomarkers to differentiate malignant thyroid nodules with cytological indeterminate
Source: BMC Cancer. 2020 Mar 12;20:199. doi: 10.1186/s12885-020-6676-z (PMC7066786; doi:10.1186/s12885-020-6676-z)
Supplement: Supplementary file 7 — Additional file 7: Table S1. The samples information and case group sorting. [file 12885_2020_6676_MOESM7_ESM.pdf]

Supporting Table.1 Samples information of different case groups.

Case groups	Sample ID	Cytology	Histopathology	Age (yrs)
	GSM846410	indeterminate	benign	59
	GSM846411	indeterminate	benign	31
	GSM846413	indeterminate	benign	64
	GSM846414	indeterminate	benign	63
	GSM846415	indeterminate	benign	47
	GSM846422	indeterminate	benign	44
	GSM846423	indeterminate	benign	38
	GSM846424	indeterminate	benign	55
	GSM846427	indeterminate	benign	53
	GSM846430	indeterminate	benign	36
	GSM846434	indeterminate	benign	64
	GSM846435	indeterminate	benign	62
	GSM846439	indeterminate	benign	58
	GSM846440	indeterminate	benign	25
	GSM846441	indeterminate	benign	62
	GSM846442	indeterminate	benign	67
	GSM846443	indeterminate	benign	51
	GSM846444	indeterminate	benign	56
	GSM846445	indeterminate	benign	69
	GSM846448	indeterminate	benign	69
	GSM846449	indeterminate	benign	60
	GSM846451	indeterminate	benign	47
	GSM846453	indeterminate	benign	46
	GSM846454	indeterminate	benign	37
	GSM846455	indeterminate	benign	40
	GSM846456	indeterminate	benign	55
	GSM846460	indeterminate	benign	55
	GSM846462	indeterminate	benign	55
	GSM846463	indeterminate	benign	56
	GSM846464	indeterminate	benign	44
	GSM846465	indeterminate	benign	29
	GSM846466	indeterminate	benign	43
	GSM846467	indeterminate	benign	50
	GSM846470	indeterminate	benign	36
	GSM846471	indeterminate	benign	48
	GSM846472	indeterminate	benign	66
	GSM846473	indeterminate	benign	68
	GSM846474	indeterminate	benign	37
	GSM846487	indeterminate	benign	66
	GSM846488	indeterminate	benign	51
	GSM846492	indeterminate	benign	70
	GSM846493	indeterminate	benign	53
	GSM846498	indeterminate	benign	70
	GSM846499	indeterminate	benign	59
	GSM846500	indeterminate	benign	62
	GSM846504	indeterminate	benign	61
	GSM846507	indeterminate	benign	50

	GSM846508	indeterminate	benign	35
	GSM846509	indeterminate	benign	64
	GSM846510	indeterminate	benign	37
	GSM846511	indeterminate	benign	66
	GSM846513	indeterminate	benign	64
	GSM846515	indeterminate	benign	48
	GSM846517	indeterminate	benign	52
	GSM846519	indeterminate	benign	73
	GSM846520	indeterminate	benign	52
	GSM846526	indeterminate	benign	81
	GSM846527	indeterminate	benign	37
	GSM846528	indeterminate	benign	48
	GSM846529	indeterminate	benign	44
	GSM846535	indeterminate	benign	57
	GSM846537	indeterminate	benign	54
	GSM846538	indeterminate	benign	65
	GSM846543	indeterminate	benign	54
	GSM846550	indeterminate	benign	49
	GSM846551	indeterminate	benign	56
	GSM846552	indeterminate	benign	52
	GSM846553	indeterminate	benign	49
	GSM846555	indeterminate	benign	34
	GSM846556	indeterminate	benign	26
	GSM846563	indeterminate	benign	35
	GSM846564	indeterminate	benign	51
	GSM846565	indeterminate	benign	43
	GSM846567	indeterminate	benign	63
	GSM846569	indeterminate	benign	52
	GSM846573	indeterminate	benign	48
	GSM846574	indeterminate	benign	45
	GSM846578	indeterminate	benign	31
	GSM846579	indeterminate	benign	58
	GSM846585	indeterminate	benign	69
	GSM846586	indeterminate	benign	75
	GSM846587	indeterminate	benign	44
	GSM846588	indeterminate	benign	60
	GSM846591	indeterminate	benign	43
	GSM846595	indeterminate	benign	59
	GSM846596	indeterminate	benign	33
	GSM846597	indeterminate	benign	39
	GSM846600	indeterminate	benign	45
	GSM846602	indeterminate	benign	69
	GSM846603	indeterminate	benign	44
	GSM846604	indeterminate	benign	56
	GSM846605	indeterminate	benign	65
	GSM846607	indeterminate	benign	56
	GSM846610	indeterminate	benign	59
	GSM846611	indeterminate	benign	33
	GSM846614	indeterminate	benign	71
	GSM846615	indeterminate	benign	41
	GSM846616	indeterminate	benign	47

	GSM846617	indeterminate	benign	46
	GSM846619	indeterminate	benign	68
	GSM846620	indeterminate	benign	45
	GSM846621	indeterminate	benign	34
	GSM846624	indeterminate	benign	58
	GSM846626	indeterminate	benign	58
	GSM846629	indeterminate	benign	75
	GSM846630	indeterminate	benign	53
	GSM846631	indeterminate	benign	68
	GSM846632	indeterminate	benign	22
	GSM846634	indeterminate	benign	62
	GSM846635	indeterminate	benign	82
	GSM846636	indeterminate	benign	48
	GSM846637	indeterminate	benign	26
	GSM846639	indeterminate	benign	39
	GSM846641	indeterminate	benign	78
	GSM846643	indeterminate	benign	54
	GSM846644	indeterminate	benign	58
	GSM846646	indeterminate	benign	79
	GSM846648	indeterminate	benign	52
	GSM846650	indeterminate	benign	26
	GSM846651	indeterminate	benign	50
	GSM846652	indeterminate	benign	42
	GSM846653	indeterminate	benign	46
	GSM846655	indeterminate	benign	71
	GSM846656	indeterminate	benign	59
	GSM846657	indeterminate	benign	62
	GSM846659	indeterminate	benign	84
	GSM846660	indeterminate	benign	45
	GSM846661	indeterminate	benign	37
	GSM846662	indeterminate	benign	40
	GSM846664	indeterminate	benign	37
	GSM846665	indeterminate	benign	46
One	GSM846666	indeterminate	benign	61
	GSM846667	indeterminate	benign	53
	GSM846670	indeterminate	benign	60
	GSM846673	indeterminate	benign	56
	GSM846677	indeterminate	benign	46
	GSM846678	indeterminate	benign	33
	GSM846681	indeterminate	benign	58
	GSM846682	indeterminate	benign	38
	GSM846686	indeterminate	benign	61
	GSM846688	indeterminate	benign	47
	GSM846690	indeterminate	benign	67
	GSM846691	indeterminate	benign	71
	GSM846692	indeterminate	benign	38
	GSM846693	indeterminate	benign	63
	GSM846699	indeterminate	benign	59
	GSM846700	indeterminate	benign	50
	GSM846704	indeterminate	benign	24
	GSM846705	indeterminate	benign	37

	GSM846707	indeterminate	benign	50
	GSM846708	indeterminate	benign	51
	GSM846709	indeterminate	benign	38
	GSM846712	indeterminate	benign	59
	GSM846714	indeterminate	benign	52
	GSM846715	indeterminate	benign	76
	GSM846716	indeterminate	benign	53
	GSM846720	indeterminate	benign	58
	GSM846723	indeterminate	benign	47
	GSM846724	indeterminate	benign	42
	GSM846726	indeterminate	benign	51
	GSM846727	indeterminate	benign	58
	GSM846729	indeterminate	benign	50
	GSM846730	indeterminate	benign	33
	GSM846731	indeterminate	benign	41
	GSM846732	indeterminate	benign	65
	GSM846736	indeterminate	benign	49
	GSM846743	indeterminate	benign	39
	GSM846744	indeterminate	benign	45
	GSM846746	indeterminate	benign	50
	GSM846747	indeterminate	benign	37
	GSM846749	indeterminate	benign	79
	GSM846751	indeterminate	benign	61
	GSM846752	indeterminate	benign	50
	GSM846754	indeterminate	benign	60
	GSM846761	indeterminate	benign	53
	GSM846769	indeterminate	benign	68
	GSM846771	indeterminate	benign	25
	GSM846772	indeterminate	benign	73
	GSM846775	indeterminate	benign	46
	GSM846777	indeterminate	benign	65
	GSM846417	indeterminate	malignant	58
	GSM846418	indeterminate	malignant	85
	GSM846425	indeterminate	malignant	62
	GSM846428	indeterminate	malignant	28
	GSM846432	indeterminate	malignant	56
	GSM846436	indeterminate	malignant	61
	GSM846438	indeterminate	malignant	42
	GSM846447	indeterminate	malignant	45
	GSM846452	indeterminate	malignant	41
	GSM846458	indeterminate	malignant	55
	GSM846478	indeterminate	malignant	66
	GSM846479	indeterminate	malignant	28
	GSM846481	indeterminate	malignant	39
	GSM846486	indeterminate	malignant	37
	GSM846490	indeterminate	malignant	66
	GSM846494	indeterminate	malignant	65
	GSM846495	indeterminate	malignant	39
	GSM846497	indeterminate	malignant	55
	GSM846501	indeterminate	malignant	51
	GSM846503	indeterminate	malignant	39

	GSM846512	indeterminate	malignant	53
	GSM846521	indeterminate	malignant	39
	GSM846524	indeterminate	malignant	23
	GSM846525	indeterminate	malignant	46
	GSM846530	indeterminate	malignant	51
	GSM846531	indeterminate	malignant	74
Two	GSM846532	indeterminate	malignant	38
	GSM846533	indeterminate	malignant	43
	GSM846536	indeterminate	malignant	50
	GSM846539	indeterminate	malignant	30
	GSM846540	indeterminate	malignant	54
	GSM846544	indeterminate	malignant	44
	GSM846545	indeterminate	malignant	31
	GSM846547	indeterminate	malignant	62
	GSM846548	indeterminate	malignant	36
	GSM846568	indeterminate	malignant	45
	GSM846570	indeterminate	malignant	39
	GSM846571	indeterminate	malignant	33
	GSM846575	indeterminate	malignant	69
	GSM846576	indeterminate	malignant	51
	GSM846577	indeterminate	malignant	53
	GSM846580	indeterminate	malignant	34
	GSM846581	indeterminate	malignant	24
	GSM846583	indeterminate	malignant	49
	GSM846589	indeterminate	malignant	41
	GSM846590	indeterminate	malignant	48
	GSM846593	indeterminate	malignant	46
	GSM846601	indeterminate	malignant	56
	GSM846606	indeterminate	malignant	49
	GSM846613	indeterminate	malignant	39
	GSM846618	indeterminate	malignant	52
	GSM846623	indeterminate	malignant	46
	GSM846627	indeterminate	malignant	46
	GSM846628	indeterminate	malignant	77
	GSM846645	indeterminate	malignant	57
	GSM846647	indeterminate	malignant	52
	GSM846663	indeterminate	malignant	45
	GSM846668	indeterminate	malignant	61
	GSM846676	indeterminate	malignant	42
	GSM846679	indeterminate	malignant	43
	GSM846683	indeterminate	malignant	54
	GSM846689	indeterminate	malignant	53
	GSM846694	indeterminate	malignant	60
	GSM846695	indeterminate	malignant	55
	GSM846697	indeterminate	malignant	73
	GSM846703	indeterminate	malignant	59
	GSM846706	indeterminate	malignant	57
	GSM846710	indeterminate	malignant	66
	GSM846713	indeterminate	malignant	37
	GSM846717	indeterminate	malignant	59
	GSM846719	indeterminate	malignant	46

	GSM846721	indeterminate	malignant	57
	GSM846733	indeterminate	malignant	34
	GSM846734	indeterminate	malignant	52
	GSM846735	indeterminate	malignant	72
	GSM846738	indeterminate	malignant	59
	GSM846739	indeterminate	malignant	31
	GSM846745	indeterminate	malignant	71
	GSM846753	indeterminate	malignant	66
	GSM846757	indeterminate	malignant	48
	GSM846762	indeterminate	malignant	38
	GSM846765	indeterminate	malignant	31
	GSM846767	indeterminate	malignant	51
	GSM846768	indeterminate	malignant	36
	GSM846773	indeterminate	malignant	58
	GSM846416	benign	benign	42
	GSM846431	benign	benign	54
	GSM846446	benign	benign	66
	GSM846457	benign	benign	51
	GSM846468	benign	benign	57
	GSM846469	benign	benign	67
	GSM846477	benign	benign	53
	GSM846480	benign	benign	74
	GSM846482	benign	benign	48
	GSM846484	benign	benign	48
	GSM846505	benign	benign	45
	GSM846506	benign	benign	65
	GSM846514	benign	benign	47
	GSM846516	benign	benign	62
	GSM846518	benign	benign	51
	GSM846534	benign	benign	53
	GSM846542	benign	benign	56
	GSM846546	benign	benign	48
	GSM846554	benign	benign	66
Three	GSM846559	benign	benign	44
	GSM846562	benign	benign	57
	GSM846572	benign	benign	57
	GSM846584	benign	benign	46
	GSM846592	benign	benign	42
	GSM846612	benign	benign	52
	GSM846625	benign	benign	61
	GSM846633	benign	benign	54
	GSM846638	benign	benign	61
	GSM846658	benign	benign	30
	GSM846669	benign	benign	35
	GSM846672	benign	benign	60
	GSM846680	benign	benign	48
	GSM846698	benign	benign	51
	GSM846701	benign	benign	55
	GSM846718	benign	benign	60
	GSM846722	benign	benign	39
	GSM846728	benign	benign	77

	GSM846741	benign	benign	56
	GSM846748	benign	benign	56
	GSM846755	benign	benign	57
	GSM846756	benign	benign	58
	GSM846764	benign	benign	50
	GSM846766	benign	benign	60
	GSM846774	benign	benign	61
	GSM846412	malignant	malignant	47
	GSM846419	malignant	malignant	29
	GSM846420	malignant	malignant	40
	GSM846421	malignant	malignant	22
	GSM846426	malignant	malignant	43
	GSM846429	malignant	malignant	60
	GSM846433	malignant	malignant	80
	GSM846437	malignant	malignant	33
	GSM846450	malignant	malignant	30
	GSM846459	malignant	malignant	22
	GSM846461	malignant	malignant	35
	GSM846475	malignant	malignant	74
	GSM846476	malignant	malignant	74
	GSM846483	malignant	malignant	49
	GSM846485	malignant	malignant	25
	GSM846489	malignant	malignant	49
	GSM846491	malignant	malignant	56
	GSM846496	malignant	malignant	64
	GSM846502	malignant	malignant	30
	GSM846522	malignant	malignant	34
	GSM846523	malignant	malignant	67
	GSM846541	malignant	malignant	47
	GSM846549	malignant	malignant	43
	GSM846557	malignant	malignant	45
	GSM846558	malignant	malignant	39
Four	GSM846560	malignant	malignant	38
	GSM846561	malignant	malignant	49
	GSM846582	malignant	malignant	57
	GSM846594	malignant	malignant	62
	GSM846598	malignant	malignant	53
	GSM846599	malignant	malignant	33
	GSM846609	malignant	malignant	36
	GSM846640	malignant	malignant	26
	GSM846642	malignant	malignant	46
	GSM846649	malignant	malignant	59
	GSM846654	malignant	malignant	71
	GSM846671	malignant	malignant	28
	GSM846674	malignant	malignant	42
	GSM846675	malignant	malignant	31
	GSM846684	malignant	malignant	38
	GSM846685	malignant	malignant	78
	GSM846696	malignant	malignant	43
	GSM846702	malignant	malignant	48
	GSM846711	malignant	malignant	65

	GSM846725	malignant	malignant	35
	GSM846737	malignant	malignant	49
	GSM846740	malignant	malignant	48
	GSM846742	malignant	malignant	60
	GSM846750	malignant	malignant	64
	GSM846758	malignant	malignant	67
	GSM846759	malignant	malignant	50
	GSM846760	malignant	malignant	38
	GSM846763	malignant	malignant	48
	GSM846770	malignant	malignant	50
	GSM846776	malignant	malignant	70
