# Supplementary material for: Identification of potential novel biomarkers to differentiate malignant thyroid nodules with cytological indeterminate
Source: BMC Cancer. 2020 Mar 12;20:199. doi: 10.1186/s12885-020-6676-z (PMC7066786; doi:10.1186/s12885-020-6676-z)
Supplement: Supplementary file 8 — Additional file 8: Table S2. The pick soft threshold for Module. [file 12885_2020_6676_MOESM8_ESM.pdf]

Supporting Table.2 The pick soft threshold for Module

	Power	SFT.R.sq	slope	truncated.R.sq	mean.k.	median.k.	max.k.
1	1	2.05E-01	0.521	0.73	42.9	4.13E+01	71.3
2	2	2.95E-01	-0.397	0.767	17.1	1.49E+01	40.5
3	3	6.57E-01	-0.692	0.766	8.67	6.59E+00	26.8
4	4	8.23E-01	-0.981	0.779	5.09	3.32E+00	19.4
5	5	8.59E-01	-1.03	0.831	3.31	1.83E+00	14.8
6	6	7.47E-01	-1.08	0.726	2.32	1.08E+00	12
7	7	8.14E-01	-1.03	0.83	1.71	7.05E-01	9.96
8	8	7.91E-01	-1.09	0.827	1.31	4.89E-01	8.43
9	9	7.45E-01	-1.06	0.782	1.04	3.22E-01	7.21
10	10	7.14E-01	-1.02	0.775	0.841	2.26E-01	6.24
11	12	8.13E-01	-1.02	0.849	0.583	1.18E-01	4.83
12	14	8.41E-01	-0.968	0.862	0.426	5.81E-02	3.88
13	16	8.89E-01	-0.929	0.902	0.322	2.76E-02	3.17
14	18	8.33E-01	-0.938	0.804	0.25	1.34E-02	2.62
15	20	1.73E-01	-1.63	-0.0414	0.198	7.00E-03	2.18
