# Supplementary material for: Identification of potential novel biomarkers to differentiate malignant thyroid nodules with cytological indeterminate
Source: BMC Cancer. 2020 Mar 12;20:199. doi: 10.1186/s12885-020-6676-z (PMC7066786; doi:10.1186/s12885-020-6676-z)
Supplement: Supplementary file 9 — Additional file 9: Table S3. List of the 29 Genes in the grey module. [file 12885_2020_6676_MOESM9_ESM.pdf]

Supporting Table 3. List of the 29 Genes in the grey module.

ProbeID	GeneSymbol	mRNAAccession	GS	p.GS.histology	MM.grey	p.MM.grey
3428225	NR1H4	NM_005123	-0.23	1.26E-04	0.65	1.88E-33
2585400	SCN9A	NM_002977	-0.22	3.32E-04	0.16	9.30E-03
2430163	VTCN1	NM_024626	0.22	3.72E-04	0.17	7.27E-03
3484895	KL	NM_004795	0.21	4.85E-04	0.16	8.55E-03
2739308	EGF	NM_001963	-0.21	6.20E-04	0.36	1.51E-09
2981874	DYNLT1	NM_006519	-0.19	1.82E-03	0.13	2.86E-02
3811086	PIGN	NM_176787	-0.19	2.31E-03	-0.49	1.23E-17
3006572	AUTS2	NM_015570	0.18	3.92E-03	0.34	2.55E-08
3142381	FABP4	NM_001442	-0.16	9.41E-03	-0.09	1.53E-01
3393479	FXYD6	NM_001164836	-0.15	1.22E-02	-0.01	8.52E-01
3159754	DMRT2	NM_006557	-0.12	5.19E-02	0.41	8.29E-12
3343832	TYR	NM_000372	-0.11	6.73E-02	0.75	4.13E-48
3264997	C10orf81	NM_001193434	-0.11	6.84E-02	0.68	7.57E-37
2894790	SYCP2L	NM_001040274	-0.09	1.27E-01	0.55	1.26E-22
2372812	RGS13	NM_002927	0.09	1.37E-01	-0.05	4.51E-01
3363686	KIDINS220	NM_020738	-0.08	2.05E-01	0.25	3.18E-05
2730303	C4orf7	NM_152997	0.07	2.30E-01	-0.06	3.13E-01
2490351	CTNNA2	NM_004389	-0.07	2.43E-01	0.86	1.67E-79
2732508	CXCL13	NM_006419	0.06	3.57E-01	-0.15	1.48E-02
3364127	CALCA	NM_001741	-0.06	3.59E-01	0.47	7.87E-16
2479698	SLC3A1	NM_000341	-0.05	3.91E-01	-0.08	1.78E-01
3242353	CREM	NM_183013	-0.05	4.45E-01	-0.22	4.32E-04
3039830	AGR3	NM_176813	0.05	4.53E-01	0.32	1.53E-07
3811949	CDH19	NM_021153	-0.04	5.14E-01	0.82	2.53E-65
2941690	GCM2	NM_004752	-0.04	5.51E-01	0.72	1.03E-43
3834341	CEACAM5	NM_004363	-0.04	5.69E-01	0.33	3.43E-08
3214845	ASPN	NM_017680	0.03	6.40E-01	0.09	1.34E-01
3457336	SILV	NM_006928	0.02	7.53E-01	0.11	8.78E-02
2923928	FABP7	NM_001446	-0.01	8.42E-01	0.51	1.70E-18
