# Supplementary material for: Identification of potential novel biomarkers to differentiate malignant thyroid nodules with cytological indeterminate
Source: BMC Cancer. 2020 Mar 12;20:199. doi: 10.1186/s12885-020-6676-z (PMC7066786; doi:10.1186/s12885-020-6676-z)
Supplement: Supplementary file 10 — Additional file 10: Table S4. List of the 24 significant genes in the blue module. [file 12885_2020_6676_MOESM10_ESM.pdf]

Supporting Table.4 List of the 24 Genes in the blue module.

ProbeID	GeneSymbol	mRNAAccession	GS	p.GS.histology	MM.blue	p.MM.blue
2708855	LIPH	NM_139248	0.5473	5.94E-22	0.9091	3.15E-101
2884845	GABRB2	NM_021911	0.5261	4.02E-20	0.8046	5.11E-61
3329343	MDK	NM_001012334	0.5186	1.66E-19	0.6857	7.26E-38
2657808	CLDN16	NM_006580	0.5088	1.01E-18	0.8588	8.64E-78
2710599	CLDN1	NM_021101	0.4566	5.95E-15	0.8675	3.91E-81
2442008	RXRG	NM_006917	0.4530	1.04E-14	0.8209	1.84E-65
3757108	KRT19	NM_002276	0.4359	1.27E-13	0.7275	1.35E-44
4012178	CITED1	NM_001144885	0.4347	1.50E-13	0.8393	4.94E-71
2685304	PROS1	NM_000313	0.4295	3.15E-13	0.8289	8.28E-68
3494629	SCEL	NM_144777	0.4073	6.27E-12	0.7385	1.43E-46
2721959	SLC34A2	NM_001177999	0.4041	9.42E-12	0.7620	3.56E-51
2562529	ST3GAL5	NM_003896	0.3967	2.40E-11	0.8109	1.08E-62
3338192	CCND1	NM_053056	0.3864	8.61E-11	0.8098	2.26E-62
2396750	FBXO2	NM_012168	0.3740	3.71E-10	0.5680	7.16E-24
4020655	ODZ1	NM_001163278	0.3604	1.74E-09	0.7082	2.42E-41
2834282	STK32A	NM_001112724	0.3457	8.47E-09	0.7486	1.70E-48
2598261	FN1	NM_212482	0.3252	6.82E-08	0.5546	1.31E-22
3212008	FRMD3	NM_174938	0.3208	1.05E-07	0.7488	1.59E-48
2378068	G0S2	NM_015714	0.2965	9.78E-07	0.3430	1.13E-08
3365136	SERGEF	NM_012139	0.2903	1.67E-06	0.6219	1.54E-29
3259367	CC2D2B	NM_001159747	0.2628	1.57E-05	0.5396	2.84E-21
2373336	CFH	NM_000186	0.2590	2.11E-05	0.4242	6.52E-13
3341497	NDUFC2	NM_004549	0.2461	5.49E-05	0.5640	1.74E-23
2880051	PPP2R2B	NM_181674	-0.2188	3.51E-04	-0.5678	7.53E-24
