# Supplementary material for: Identification of potential novel biomarkers to differentiate malignant thyroid nodules with cytological indeterminate
Source: BMC Cancer. 2020 Mar 12;20:199. doi: 10.1186/s12885-020-6676-z (PMC7066786; doi:10.1186/s12885-020-6676-z)
Supplement: Supplementary file 11 — Additional file 11: Table S5. The Copy-number Alterations of significant genes. [file 12885_2020_6676_MOESM11_ESM.pdf]

Supporting Table. 5 Copy-number Alterations of Significant Genes .

Gene Symbol	OQL Line	Num Samples Altered	Percent Samples Altered
SCEL	SCEL: AMP HOMDEL MUT FUSION;	8	0.009
CFH	CFH: AMP HOMDEL MUT FUSION;	7	0.008
G0S2	G0S2: AMP HOMDEL MUT FUSION;	7	0.008
FN1	FN1: AMP HOMDEL MUT FUSION;	4	0.005
PROS1	PROS1: AMP HOMDEL MUT FUSION;	4	0.005
TENM1	TENM1: AMP HOMDEL MUT FUSION;	3	0.003
RXRG	RXRG: AMP HOMDEL MUT FUSION;	3	0.003
STK32A	STK32A: AMP HOMDEL MUT FUSION;	3	0.003
CC2D2B	CC2D2B: AMP HOMDEL MUT FUSION;	2	0.002
GABRB2	GABRB2: AMP HOMDEL MUT FUSION;	2	0.002
KRT19	KRT19: AMP HOMDEL MUT FUSION;	2	0.002
PPP2R2B	PPP2R2B: AMP HOMDEL MUT FUSION;	2	0.002
SERGEF	SERGEF: AMP HOMDEL MUT FUSION;	2	0.002
SLC34A2	SLC34A2: AMP HOMDEL MUT FUSION;	2	0.002
CITED1	CITED1: AMP HOMDEL MUT FUSION;	1	0.001
