# Supplementary material for: Identification of potential novel biomarkers to differentiate malignant thyroid nodules with cytological indeterminate
Source: BMC Cancer. 2020 Mar 12;20:199. doi: 10.1186/s12885-020-6676-z (PMC7066786; doi:10.1186/s12885-020-6676-z)
Supplement: Supplementary file 12 — Additional file 12: Table S6. The Mutual Exclusivity tab of significant gene pairs. The genes pairs alternated in Thyroid cancer are mutual exclusivity. The tab provides summary statistics significant on mutual exclusivity and co-occurrence of genomic alterations in each pair of query genes. The mutual exclusivity is significant for the other two gene pairs (P < 0.05). The P values are determined by a Fisher’s exact test with the null hypothesis that the frequency of occurrence of a pair of alterations in two genes is proportional to their uncorrelated occurrence in each gene. [file 12885_2020_6676_MOESM12_ESM.pdf]

Supporting Table.6 The mutual exclusivity alterations of significant Gene pairs

Gene A	Gene B	Neither	A Not B	B Not A	Both	Log Odds Ratio	p-Value	Adjusted p-Value	Tendency
**CFH**	**G0S2**	908	0	0	7	>3	<0.001	**<0.001**	Co-occurrence
**CFH**	**RXRG**	908	4	0	3	>3	<0.001	**<0.001**	Co-occurrence
**G0S2**	**RXRG**	908	4	0	3	>3	<0.001	**<0.001**	Co-occurrence
**PPP2R2B**	**STK32A**	912	0	1	2	>3	<0.001	**0.002**	Co-occurrence
**PROS1**	**SCEL**	905	2	6	2	>3	<0.001	0.11	Co-occurrence
**CITED1**	**TENM1**	912	0	2	1	>3	0.003	0.905	Co-occurrence
